# Platelet-to-lymphocyte ratio for prognostication in immune checkpoint inhibitor-treated cancer patients: a meta-analysis of 13027 patients highlighting nivolumab-responsive renal cell carcinoma

**DOI:** 10.3389/fimmu.2026.1732790

**Published:** 2026-02-02

**Authors:** Mingxing Wang, Wanhui Dong, Jian Chen, Pantong Wu, Yuru Wang, Xiaonan Zhang, Yaning Cao, Zhiying Wang, Zhixian Zhong, Yi Zhong

**Affiliations:** 1 Shanghai TCM-Integrated Hospital, Shanghai University of TCM, Shanghai, China; 2 Department of Oncology, Shanghai University of Traditional Chinese Medicine, Shanghai, China; 3 Lu’an Hospital of Traditional Chinese Medicine Affiliated to Anhui University of Chinese Medicine, Lu’an, Anhui, China; 4 Department of Oncology, Tongji University, Shanghai, China

**Keywords:** immune checkpoint inhibitor, lymphocyte, malignancy, platelet, progression-free survival

## Abstract

**Objective:**

To assess platelet-to-lymphocyte ratio (PLR) prognostic utility for overall (OS) and progression-free survival (PFS) in immune checkpoint inhibitor-treated cancer patients, and examine impacts of geography, cancer type, cutoff, ICI class, treatment line and stage.

**Methods:**

A systematic literature search identified studies investigating PLR and prognosis in ICI treated patients. Hazard ratios (HRs) with 95% confidence intervals (CIs) were pooled using random-effects models. Subgroup analyses examined key covariates; publication bias was assessed.

**Results:**

Analysis of 98 publications (86 OS, 72 PFS) demonstrated that elevated PLR was a robust predictor of shorter OS (HR 1.79, 95% CI: 1.60-2.00) and PFS (HR 1.60, 95% CI: 1.44-1.78). Subgroup analyses revealed: (1) Geographic region: Asian populations exhibited the most consistent correlation with OS and the highest PFS risk (69%). (2) Cancer type: For OS, prognostic value was maintained across all cancers; the most pronounced impacts were observed in hepatocellular carcinoma (HR 2.10), esophageal carcinoma (HR 2.08), and head and neck squamous cell carcinoma (HR 2.61). For PFS, a notable link to poor outcomes was observed in NSCLC and hepatocellular carcinoma, whereas renal cell carcinoma showed no such correlation. (3) PLR cutoff: both PLR ≥180 (OS: HR 1.87; PFS: HR 1.68) and PLR <180 (OS: HR 1.73; PFS: HR 1.53) subgroups consistently yielded unfavorable outcomes. (4) ICI category: for OS, camrelizumab showed the strongest prognostic relevance (HR 4.68), whereas for PFS, all ICIs yielded consistent results. (5) Treatment line: both first-line (OS: HR 1.98; PFS: HR 1.93) and second-line or beyond (OS: HR 1.87; PFS: HR 1.79) demonstrated clear prognostic utility without inter-subgroup differences. (6) Tumor stage: Advanced stages (III–IV, IIIB–IV, IV) confirmed the predictive value of PLR for both OS and PFS. (7) Cancer Subtypes: PLR remained prognostic in nivolumab-treated, stage IV genitourinary cancers; correlated with survival in pembrolizumab-treated but not nivolumab-treated NSCLC; and remained predictive in camrelizumab-treated/advanced gastrointestinal tumors. Notably, elevated PLR was uniquely associated with worsened OS and PFS in nivolumab-treated renal cell carcinoma.

**Conclusions:**

Elevated PLR is consistently associated with shortened OS across the cancer types receiving ICIs, while its prognostic value for PFS fluctuates depending on cancer type and ICI class. The prognostic impact of PLR is particularly robust in the nivolumab-treated RCC, pembrolizumab-treated NSCLC, camrelizumab-treated gastrointestinal tumors, and various advanced-stage malignancies.

**Systematic review registration:**

https://www.crd.york.ac.uk/prospero/.

## 1 Introduction

Over recent years, immune checkpoint inhibitors (ICIs) have become the backbone of therapy across multiple malignancies, elevating 5-year overall survival (OS) to 31.9% in advanced non-small-cell lung cancer (NSCLC) and yielding 48.3% 5-year relapse-free survival when combined with targeted therapy in BRAF-mutant melanoma (1, 2). Nevertheless, clinical practice still faces formidable challenges: the non-response rate to immunotherapy varies markedly across cancer types, reaching 30% in first-line immune-chemotherapy for advanced esophageal carcinoma, while recurrent or metastatic head and neck squamous cell carcinoma (HNSCC) achieves a median progression-free survival (PFS) of only 3.4 months with single-agent ICI; moreover, approximately 10–15% of patients develop grade ≥3 immune-related adverse events such as pneumonitis or myocarditis (3–5). Evidence indicates that inter-individual heterogeneity in inflammatory-immune homeostasis within the tumor microenvironment (TME) constitutes a central driver of therapeutic variability.

Tumor-associated inflammation, a hallmark of malignancy, is intimately linked to tumor progression and therapeutic response. Neoplastic cells, together with stromal components such as tumor-associated macrophages and cancer-associated fibroblasts, continuously release IL-6, TNF-α, GM-CSF, thereby establishing a chronic inflammatory milieu (6). Among these, IL-6 markedly impairs CD8^+^ T-cell function via JAK/STAT3 pathway activation; studies show that JAK/STAT3 signaling reduces T-cell proliferative capacity by 40–60% (7). Meanwhile, IL-6 also facilitates regulatory T-cell (Treg) differentiation; *in vitro*, Treg proportions increase approximately 2.3-fold when IL-6 exceeds 10 pg/mL, thereby intensifying immunosuppression (8). Under tumor-associated inflammation, the infiltrating fraction of M2-polarized tumor-associated macrophages can reach 60% of total TAMs, a proportion more than ten-fold higher than that in adjacent non-malignant tissues (9). These cells directly suppress effector T-cell function via TGF-β secretion, and their density correlates inversely with ICI response (10). Upregulation of vascular endothelial growth factor (VEGF) driven by inflammatory cytokines represents a hallmark of tumor-associated inflammation. Serum VEGF levels in patients with advanced malignancies are elevated, with mean concentrations approximately 5.8-fold higher than those in healthy individuals (11). VEGF not only promotes tumor angiogenesis but also impedes lymphocyte infiltration into the tumor core by forming a vascular barrier, thereby compromising the potential efficacy of immunotherapy (12).

The platelet-to-lymphocyte ratio (PLR) is a composite index that has received considerable attention in recent years. It is calculated as the platelet count divided by the absolute lymphocyte count, and can reflect information on both inflammation and tumor immunity. On one hand, GM-CSF in the tumor microenvironment can activate the aryl hydrocarbon receptor (AHR) pathway in macrophages, upregulate thrombopoietin (TPO) expression, and increase the peripheral blood platelet count (13). Activated platelets not only exacerbate immunosuppression by releasing IL-6 and TGF-β but also bind to CD44 on tumor cell surfaces, thereby promoting tumor metastasis (14). On the other hand, the persistent inflammatory state induces lymphocyte apoptosis and increases the proportion of exhausted CD8^+^ T-cell subsets, thereby weakening the anti-tumor immune response (15).

However, the clinical predictive value of PLR remains controversial. In lung cancer, a 2019 retrospective study demonstrated that patients with PLR ≥180 had a median progression-free survival (mPFS) that was 4.4 months shorter than those with PLR <180, with an overall survival (OS) hazard ratio (HR) of 2.239 (95% CI: 1.478-3.392). In contrast, a single-center retrospective study focusing on BRAF wild-type metastatic melanoma found no such association; in that study, PLR showed no statistical correlation with patient OS (HR = 1.00, 95% CI: 0.99-1.01, P = 0.87) and demonstrated no prognostic value in either univariate or multivariate analysis (16, 17). The core of this discrepancy lies in the fact that PLR’s predictive efficacy is influenced by multiple factors. First, differences in cancer types and tumor microenvironment (TME) contribute to varied immunologic contexts across malignancies, leading to inconsistent associations with PLR. Second, therapeutic regimens and types of immune checkpoint inhibitors (ICIs) play a role: treatments such as ICI monotherapy versus combinations with chemotherapy or anti-angiogenic agents may alter PLR baseline levels, while distinct mechanisms of anti-PD-1/PD-L1 versus anti-CTLA-4 agents may lead to differential interactions with PLR. Third, variations in demographic and geographic factors—including genetic background, baseline immune status, and treatment strategies between Eastern and Western populations-may result in divergent PLR cutoff selections and predictive performance. Therefore, this study aims to systematically evaluate and perform a meta-analysis to clarify the overall impact of PLR on survival outcomes in cancer patients receiving immunotherapy, while using subgroup analyses to examine whether cancer type, treatment strategy, ICI class, and geographic region represent sources of heterogeneity, ultimately providing comprehensive evidence to support the clinical application of PLR.

## 2 Materials and methods

### 2.1 Study design and registration

This study is a systematic review and meta-analysis based on clinical research. The study protocol was registered on the PROSPERO international prospective register of systematic reviews (Registration ID: CRD420251171930). There were no substantive differences between the registered content and the final implementation process, ensuring research transparency and reproducibility.

### 2.2 Literature search strategy

#### 2.2.1 Databases and time scope

A comprehensive search was conducted across four electronic databases: PubMed, Embase, the Cochrane Library, and Web of Science. The search timeframe spanned from the inception of each database to October 20, 2025.

#### 2.2.2 Search terms

A combination of subject headings and free-text terms was employed. The English search terms included: “platelet-to-lymphocyte ratio”, “PLR”, “immune checkpoint inhibitor”, “ICI”, “PD-1 inhibitor”, “PD-L1 inhibitor”, “CTLA-4 inhibitor”, “cancer”, “carcinoma”, “malignancy”, “prognosis”, “survival”, “overall survival”, “OS”, “progression-free survival”, “PFS”, among others. The detailed search strategy is available in **Supplementary Table S1**.

### 2.3 Literature inclusion and exclusion criteria

#### 2.3.1 Inclusion criteria

(1) Study type: Prospective cohort studies, retrospective cohort studies, or case-control studies (only those focusing on prognostic analysis in patients receiving ICI therapy were included); (2) Study population: Patients with pathologically confirmed malignant tumors who received at least one cycle of ICI treatment (including anti-PD-1/PD-L1 monotherapy, anti-CTLA-4 monotherapy, or dual immunotherapy); (3) Exposure factor: Baseline PLR measured before treatment (within 1–2 weeks prior to the first ICI administration), with a clearly reported PLR cutoff value; (4) Outcome measures: Reporting of at least one key survival outcome (OS or PFS) as a hazard ratio with 95% confidence intervals; (5) Publication language: Only studies published in English were included.

#### 2.3.2 Exclusion criteria

(1) Studies involving non-ICI therapies; (2) Studies that did not report specific PLR values or from which HR and 95% CI could not be extracted; (3) Reviews, meta-analyses, case reports, commentaries, or laboratory-based studies; (4) Studies that did not report survival data.

### 2.4 Data extraction and quality assessment

#### 2.4.1 Data extraction

Two investigators independently extracted information using a pre-designed data extraction form. Extracted data included: first author, publication year, region, study type, sample size, cancer type, tumor stage, ICI type, and PLR cutoff value. The primary outcome was the HR and 95% CI for OS between high and low PLR groups; the secondary outcome was the HR and 95% CI for PFS between high and low PLR groups. Any discrepancies in data extraction were resolved through discussion or by a third investigator. The finalized data were managed in Excel.

#### 2.4.2 Quality assessment

The Newcastle-Ottawa Scale (NOS) was used to assess the quality of cohort studies. The NOS evaluates three domains: ‘selection of the study groups’ (4 items), ‘comparability of the groups’ (2 items), and ‘assessment of the outcome’ (3 items), with a maximum score of 9 points. Studies were categorized as follows: high-quality (NOS score ≥7), moderate-quality (NOS score 5-6), and low-quality (NOS score ≤4). Two investigators independently performed the quality assessment, with any disagreements adjudicated by a third investigator.

### 2.5 Statistical analysis

Statistical analyses were performed using R 4.3.3 and Review Manager 5.4 software. All tests were two-sided, with a statistical significance level of α=0.05. The effect measure used in this meta-analysis was the HR with its corresponding 95% CI. Prior to meta-analysis, the *χ²* test was employed to assess between-study heterogeneity (18). If *P* > 0.1 and I² < 50%, indicating no significant heterogeneity, a fixed-effects model was used to pool effect sizes; if *P* ≤ 0.1 and I² ≥ 50%, indicating significant heterogeneity, a random-effects model was applied. When significant heterogeneity was detected, sensitivity analyses were conducted to explore potential sources. To further investigate the sources of heterogeneity and identify potential moderators, univariate meta-regression analysis was performed. Subgroup analyses were conducted based on region, PLR cutoff value, cancer type, ICI class, and tumor stage. To ensure the statistical robustness of these analyses, subgroups were only evaluated if they contained at least three studies. Regarding PLR cutoff values, in addition to the aforementioned grouping criteria, the clinically commonly used threshold of 180 was also employed in supplementary analyses to validate the impact of different cutoff values on the results. Funnel plots were used to detect publication bias for clinical outcomes such as OS and PFS, supplemented by Egger’s linear regression test and Begg’s test. Furthermore, the nonparametric “trim and fill” method was utilized to evaluate the impact of potential publication bias by estimating the number of missing studies and adjusting the pooled effect sizes accordingly.

## 3 Results

### 3.1 Literature screening results

**Figure 1:**
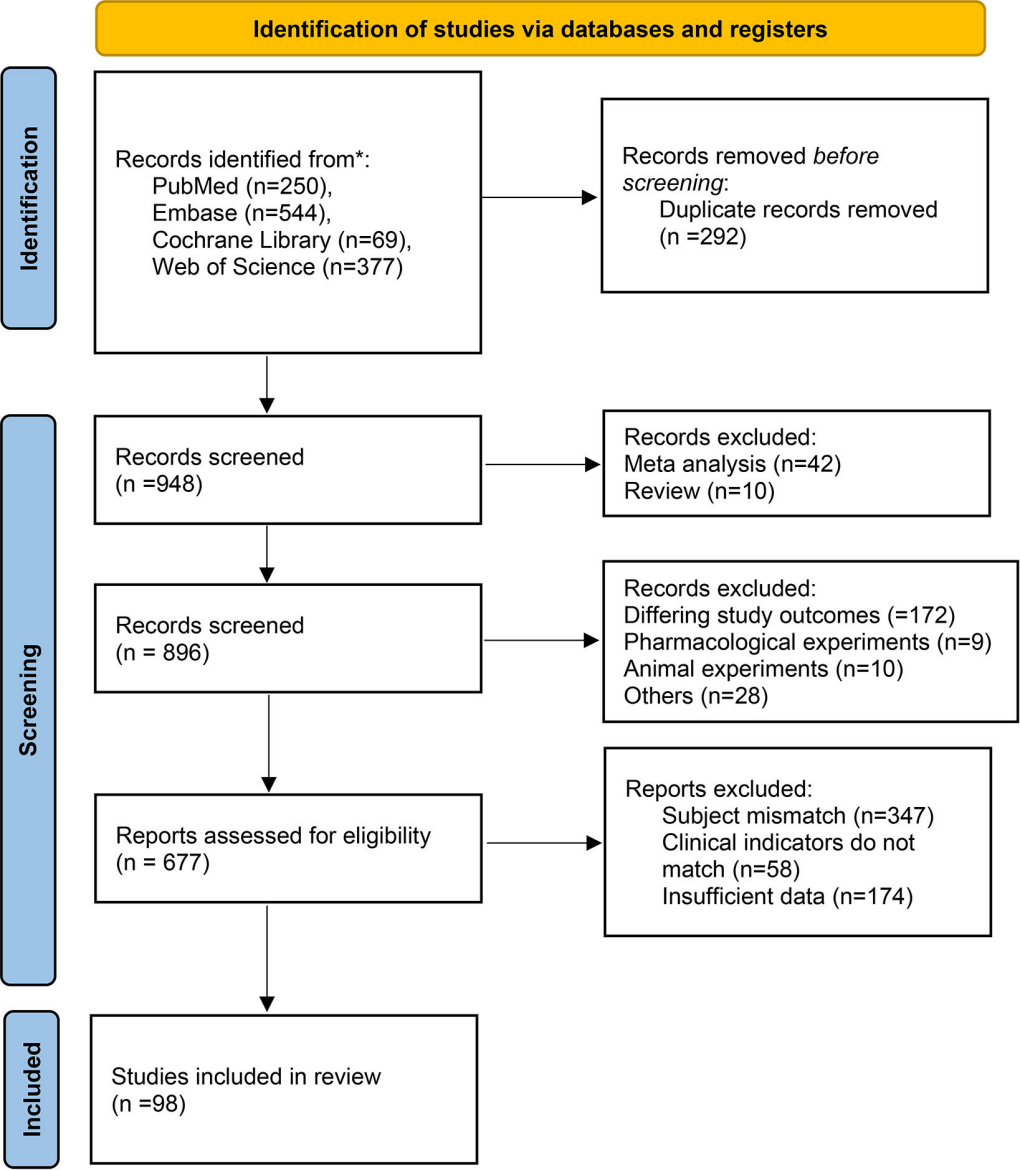
Literature search flow diagram.

**Figure 2:**
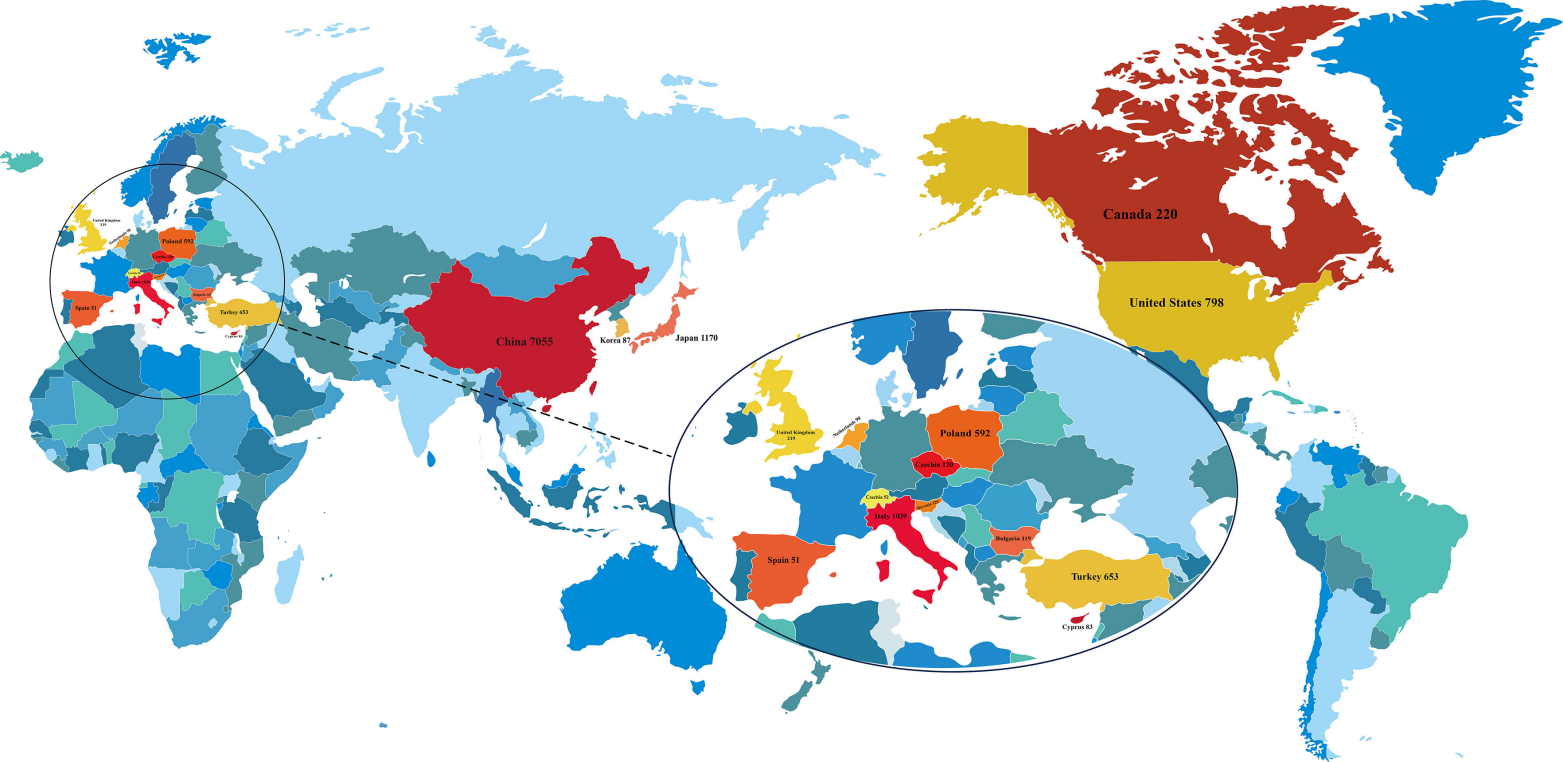
Geographical distribution of included studies.

In accordance with the PRISMA 2020 guidelines, a systematic search of the PubMed, Embase, Cochrane Library, and Web of Science databases initially identified 1240 records. After removing 292 duplicate publications, 948 records underwent title and abstract screening, which led to the exclusion of 52 articles (e.g., reviews and meta-analyses). The remaining 896 publications proceeded to full-text review. During this stage, 219 articles were excluded for the following reasons: differing study outcomes (n=172), pharmacological experiments (n=9), animal experiments (n=10), and others (n=28). Subsequently, 677 articles underwent final eligibility assessment, from which 579 were excluded due to subject mismatch (n=347), clinical indicators do not match (n=58), or insufficient data (n=174). Ultimately, 98 studies were included in the systematic review and meta-analysis (1, 19–115). The literature screening process is detailed in **Figure 1**, the distribution of the study populations is shown in **Figure 2**, and the baseline characteristics of the included studies are presented in **Table 1** (detailed baseline data are provided in **Supplementary Materials** Baseline; quality assessment results are available in **Supplementary Material S2**).

**Table 1:** Baseline characteristics of the included study populations.

Authors	Year	N	Sex (M/F)	Cutoff value	Country	Cancer type	Stage	Outcomes
Dong Hyun Kim (19)	2024	87	75/12	491.2	Korea	HNSCC	NR	OS/PFS
Ozkan Alan (20)	2025	196	167/29	196.8	Turkey	NSCLC	I-IV	OS
Volkan Aslan (21)	2023	45	31/14	206	Turkey	RCC	IV	OS/PFS
Bai R (22)	2021	103	80/23	206.5	China	Multiple	Advanced	OS
Bauckneht M (23)	2021	45	29/16	100	Italy	NSCLC	NR	OS
Mehmet A Bilen (24)	2018	90	53/37	NR	Atlanta, Georgia	Multiple	Advanced	OS/PFS
Jiaxin Cao (25)	2023	184	152/32	253.52	China	NPC	I-IV	PFS
Booka E (26)	2022	61	49/12	118	Japan	UGIC	Advanced	OS
Chen Q (27)	2024	105	NR	218.1	China	CC	Advanced	PFS
Chen X (28)	2024	200	160/40	unclear	China	HCC	BCLC B/C	OS/PFS
Chen Y (29)	2021	139	103/36	173.7	China	GC	III-IV	OS/PFS
Cheng LY (30)	2024	192	113/79	194.84	China	BCa	IV	PFS
Da L (31)	2023	162	136/26	250.51	China	ESCC	IV	OS/PFS
De Giorgi U (32)	2019	313	235/78	232	Italy	RCC	IV	OS
Dharmapuri S (33)	2020	103	86/17	500	United States	HCC	Advanced	OS
Dionese M (34)	2023	72	56/16	150	Italy	UC	IV	OS/PFS
Dong Q (35)	2024	197	160/37	154.12	China	GC	III-IV	OS
Fan X (36)	2021	111	56/55	135	China	GC and CRC	Advanced	OS/PFS
Fang Q (37)	2023	223	189/34	144.37	China	NSCLC	Advanced	OS/PFS
Gou M (38)	2022	237	109/128	139.41	China	GC	Advanced	OS/PFS
Gou M (39)	2022	186	132/54	139.41	China	GC	Advanced	OS
Guo L (40)	2024	95	79/16	150	China	NSCLC	IV	PFS
Guo Y (41)	2024	98	86/12	98.89	China	HCC	Advanced	OS/PFS
Hamai Y (42)	2023	59	52/7	270	Japan	ESCC	Advanced	OS
Hou Y (43)	2023	77	53/24	149.23	China	GC	II-IV	OS/PFS
Huai Q (44)	2023	189	159/30	157.29	China	NSCLC	I-IV	OS
Huang R (45)	2022	110	100/10	140	China	HCC	Advanced	OS/PFS
Huang X (46)	2025	133	118/15	86.7	China	HCC	BCLC B/C	OS/PFS
Iinuma K (47)	2021	35	26/9	188.1	Japan	RCC	Advanced	PFS
Ikoma T (48)	2023	93	72/21	277	Japan	ESCC	IV	OS
Inoue H (49)	2022	41	34/7	242.6	Japan	ESCC	IV	OS/PFS
Ishihara H (50)	2019	58	45/13	160	Japan	RCC	IV	OS/PFS
Jia G (51)	2023	117	106/11	131	China	HCC	BCLC B/C	OS/PFS
Jiang M (52)	2020	76	66/10	168.13	China	NSCLC	Advanced	OS/PFS
Kadono Y (53)	2021	91	65/26	181.5	Japan	UC	Advanced	OS
Katayama Y (54)	2020	81	44/37	262	Japan	NSCLC	III-IV	OS/PFS
Knetki-Wróblewska M (55)	2025	332	187/145	193.24	Poland	NSCLC	III-IV	OS/PFS
Knetki-Wróblewska M (56)	2023	260	149/111	182.9	Poland	NSCLC	Advanced	OS/PFS
Kobayashi K (57)	2025	243	179/64	252	Japan	UC	Advanced	OS
Ksienski D (58)	2021	220	99/121	441.8	Canada	NSCLC	Advanced	OS
Kurashina R (59)	2022	54	37/17	173.73	Japan	UC	Advanced	OS
Kutlu Y (60)	2023	55	42/13	135.7	Turkey	SCLC	Advanced	OS/PFS
Lei Y (61)	2025	78	51/27	195.005	China	NSCLC	NR	PFS
Li X (62)	2024	50	unclear	157.28	China	TNBC	Advanced	OS/PFS
Li Y (63)	2025	67	51/16	150.49	China	ESCC	IIIB-IV	OS/PFS
Lin X (64)	2021	87	73/14	263.76	China	Lung cancer	III-IV	OS
Liu J (65)	2022	90	56/34	157.7	China	ESCC	IIIB-IV	OS/PFS
Liu J (66)	2019	44	33/11	144	China	NSCLC	IIIB-IV	OS/PFS
Lu X (67)	2022	133	113/20	200	China	NSCLC	IIIB-IV	OS/PFS
Lu Y (68)	2025	98	87/11	140.75	China	HCC	Advanced	OS/PFS
Luo T (69)	2025	245	201/44	205.99	China	Lung cancer	Advanced	OS/PFS
Ma Y (70)	2022	95	66/29	277	China	Multiple	Advanced	PFS
Matsuki T (71)	2024	27	20/7	194.1	Japan	SGC	Advanced	PFS
Matsuo M (72)	2022	164	127/37	319.84	Japan	HNSCC	Advanced	OS
Mesti T (73)	2023	129	84/53	180	Slovenia	Melanoma	IV	OS/PFS
Mildanoglu MM (74)	2025	153	88/65	169	Turkey	GC	IV	OS/PFS
Muhammed A (75)	2021	362	284/78	300	Europe, America, Asia	HCC	BCLC A/B/C	OS/PFS
Numakura K (76)	2024	112	93/19	229	Japan	RCC	IV	OS
Olgun P (77)	2023	83	73/10	150	Republic of Cyprus	NSCLC	IIIB-IV	OS/PFS
Pan Y (78)	2021	238	176/62	163.63	China	GC	Advanced	OS/PFS
Petrova MP (79)	2020	119	74/45	200	Bulgaria	NSCLC	IV	OS
Pu D (80)	2021	184	134/50	200	China	NSCLC	IIIB-IV	OS
Qi WX (81)	2023	51	44/7	150.6	China	ESCC	II-IVA	PFS
Qi WX (82)	2021	53	34/19	119.23	China	ESCC	IV	OS
Qi Y (83)	2019	85	42/43	164	China	Multiple	Advanced	OS/PFS
Qian X (84)	2024	90	Female	190	China	TNBC	II-III	OS/PFS
Qiu X (85)	2023	67	44/23	135	China	Pancreatic cancer	Advanced	OS/PFS
Qu Z (86)	2022	106	72/34	243.33	China	GC	Advanced	OS/PFS
Rebuzzi SE (87)	2022	422	305/117	176	Italy	RCC	IV	OS/PFS
Russo A (88)	2020	187	137/50	200	Italy	NSCLC	IIIB-IV	OS
Sakai A (89)	2023	51	48/3	268	Japan	HNSCC	Advanced	OS/PFS
Sánchez-Gastaldo A (90)	2021	51	37/14	198	Spain	NSCLC	Advanced	OS/PFS
Shabto JM (91)	2020	67	53/14	301.87	United States	UC	IV	OS/PFS
Shang H (92)	2024	64	52/12	194.5	China	ESCC	III-IV	OS/PFS
Stares M (93)	2022	219	109/110	180	United Kingdom	NSCLC	Advanced	OS/PFS
Sun Y (95)	2025	142	84/58	245.65	China	GC	IV	OS/PFS
Svaton M (96)	2018	120	71/49	169.1	Czech Republic	NSCLC	Advanced	OS/PFS
Ucgul E (97)	2024	100	69/31	190.8	Turkey	Multiple	IV	OS/PFS
Ulas A (98)	2024	104	94/10	178.73	Turkey	NSCLC	Advanced	OS/PFS
Su H (94)	2024	203	158/45	305.21	China	NSCLC	III-IV	OS/PFS
Diem S (99)	2017	52	29/23	262	Switzerland	NSCLC	IV	OS/PFS
Wan M (100)	2022	45	35/10	214.08	China	GC	III-IV	OS/PFS
Wang JH (101)	2022	48	38/10	230	China	HCC	BCLC C	PFS
Willemsen ACH (102)	2023	98	83/15	241.9	Netherlands	HNSCC	Advanced	OS/PFS
Wu J (103)	2024	31	23/8	154.17	China	Gastrointestinal cancer	NR	PFS
Wu YL (104)	2022	296	245/51	300	America, Europe, Asia	HCC	BCLC C	OS/PFS
Wu Y (105)	2022	101	78/23	176	China	NSCLC	Advanced	OS/PFS
Wu Y (106)	2023	316	266/50	167.2	China	NSCLC	IIIB-IV	OS/PFS
Yang X (107)	2023	52	44/8	133	China	HCC	Advanced	OS/PFS
Yang Y (108)	2024	617	430/187	174.44	China	Gastric or Gastroesophageal junction cancer	Advanced	OS
Yang Z (109)	2022	73	49/24	222.52	China	ICC	II-IV	OS
Yildirim A (110)	2025	401	283/118	202.38	America	RCC	Advanced	OS/PFS
Yuan Q (111)	2024	107	84/23	179	China	NSCLC	IIIB-IV	OS/PFS
Zhang S (112)	2025	71	75/67	148	China	HCC	BCLC C	OS/PFS
Zhang Y (113)	2022	142	75/67	201	China	Multiple	IIIB-IV	OS/PFS
Zhao M (114)	2022	160	129/31	145.25	China	HCC	BCLC B/C	OS/PFS
Zhu M (115)	2024	88	48/40	82.23	China	GC	III-IV	OS/PFS
Zhuang TZ (1)	2025	21	Male	346.5	America	Penile Squamous Cell Carcinoma	Advanced	OS/PFS

HNSCC, Head and neck squamous cell carcinoma; RCC, Renal Cell Carcinoma; NPC, Nasopharyngeal Carcinoma; UGIC, Upper Gastrointestinal Cancer; CC, Cervical Cancer; GC, Gastric Cancer; BCa, Bladder cancer; UC, Urothelial Carcinoma; CRC, Colorectal Cancer; SGC, Salivary gland carcinoma; OS, Overall Survival; PFS, Progression-Free Survival.

### 3.2 Meta-analysis results of PLR with OS and PFS

**Figure 3:**
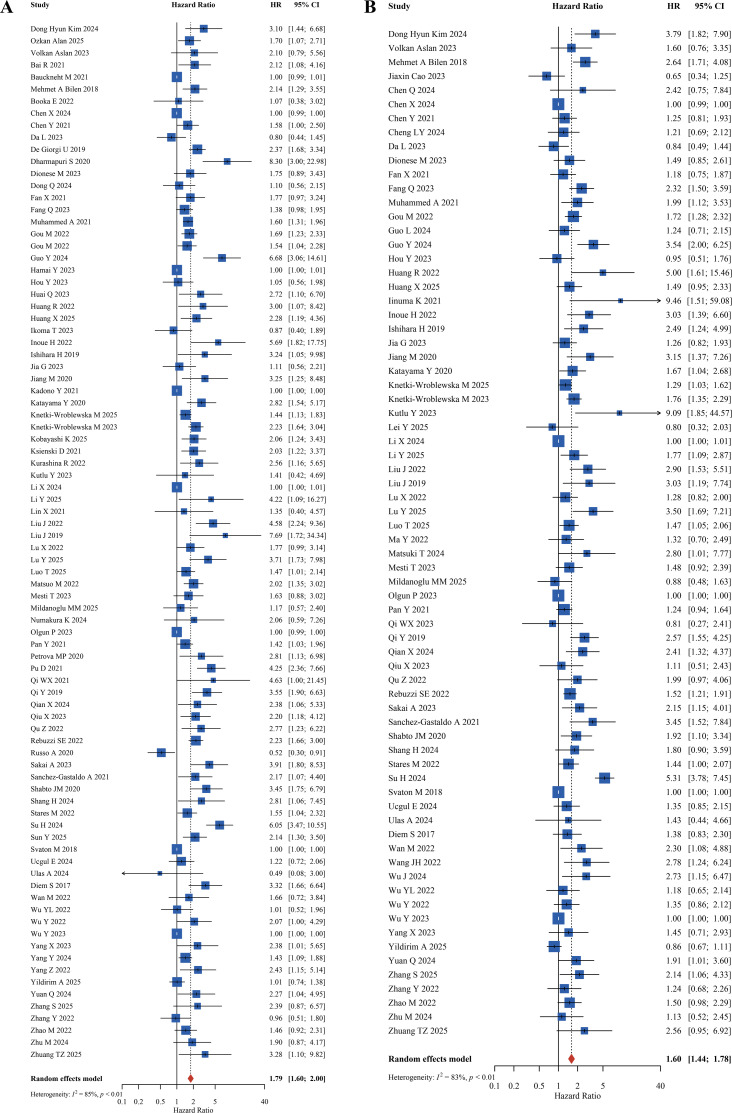
Forest plots of PLR for OS/PFS prognosis **(A)** Overall Survival (OS). **(B)** Progression-Free Survival (PFS).

Eighty-six studies reported the association between PLR levels and OS in cancer patients receiving immune checkpoint inhibitors, all providing hazard ratios and related statistics. Heterogeneity testing revealed significant heterogeneity among included studies, thus a random-effects model was applied for effect size pooling. The pooled HR was 1.79 (95% CI: 1.60-2.00, *P* < 0.01), indicating that elevated baseline PLR was significantly associated with shorter OS in cancer patients receiving immunotherapy. This suggests that higher pre-treatment PLR values are associated with a 79% increased risk of death in cancer patients undergoing immunotherapy (**Figure 3A**). Seventy-two studies reported the relationship between PLR levels and PFS in cancer patients receiving immune checkpoint inhibitors, with significant heterogeneity observed among studies. Results demonstrated that high PLR was significantly associated with reduced PFS, with a pooled HR = 1.60 (95% CI: 1.44-1.78, *P* < 0.01), indicating that high baseline PLR is also an adverse prognostic factor for PFS in cancer patients receiving immunotherapy, suggesting that higher pre-treatment PLR values increase the risk of tumor progression by 60% (**Figure 3B**).

### 3.3 Subgroup analyses of OS and PFS

#### 3.3.1 Geographic subgroup analysis

**Figure 4:**
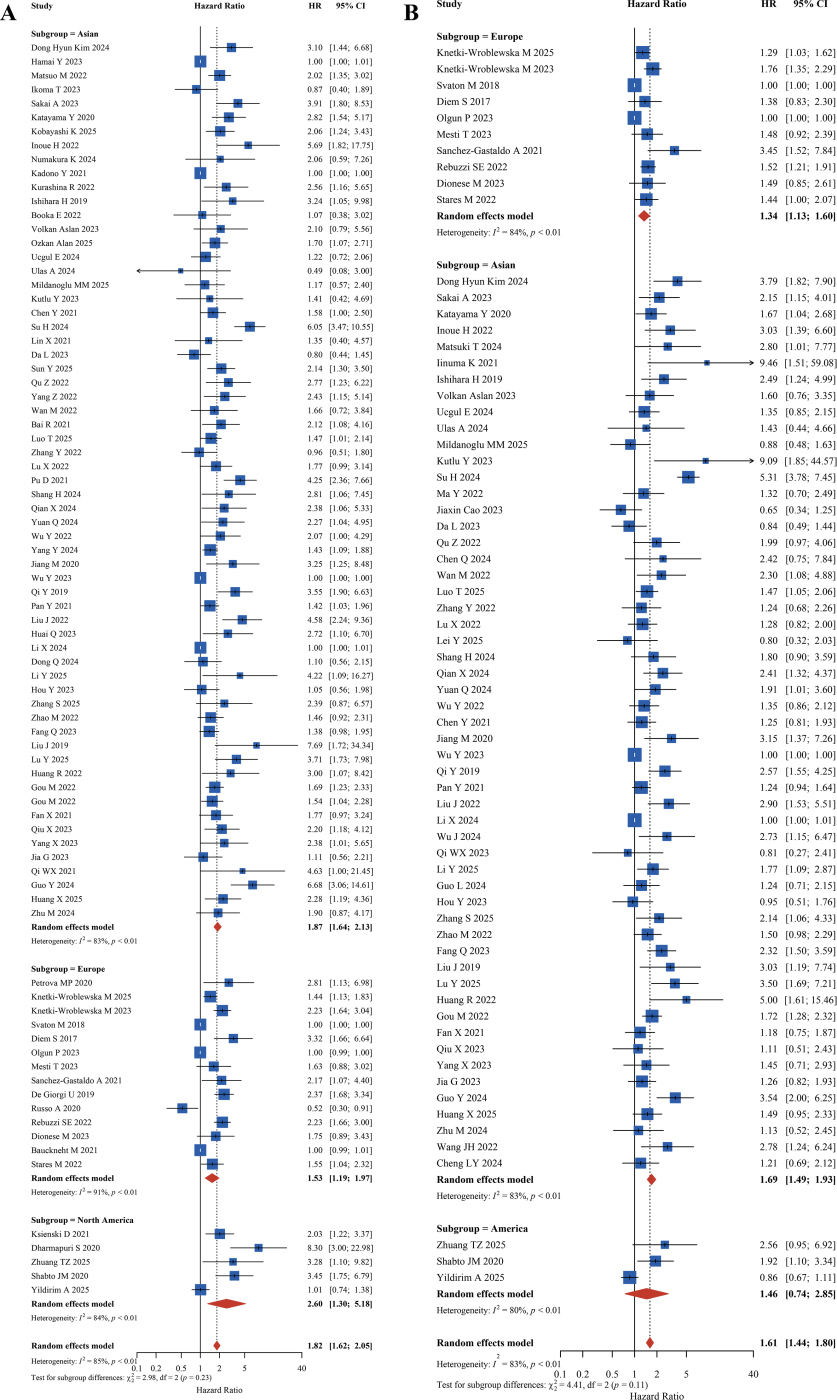
Forest plots of PLR for OS/PFS prognosis based on geographic subgroup analysis **(A)** Overall Survival (OS). **(B)** Progression-Free Survival (PFS).

Based on the geographic distribution of 82 studies, the association between PLR and OS was analyzed across three subgroups: Asia, Europe, and North America. All subgroups utilized random-effects models due to significant heterogeneity. In Asian clinical studies, most confirmed a consistent correlation between elevated PLR and shortened OS, underscoring the robust prognostic utility of PLR following immunotherapy. OS in Asian populations. Fourteen studies from Europe were included, with high within-group heterogeneity suggesting considerable fluctuation in the strength of the PLR-OS association, potentially related to variations in cancer type composition among European populations. Five studies from the Americas showed that high PLR increased mortality risk by 160% compared to low-risk groups. Overall, all three geographic subgroups indicated increased mortality risk with high PLR, though the Asian subgroup demonstrated higher reliability due to larger study numbers and greater consistency (**Figure 4A**). Among PFS studies, 68 provided geographic data on PLR’s impact, revealing that higher PLR was significantly associated with poorer PFS in Europe, Asia, and the Americas, with Asian populations showing the highest risk for disease progression at 69% (**Figure 4B**).

#### 3.3.2 Cancer type subgroup analysis

This study encompassed 10 cancer types in the OS subgroup analysis and 9 types in the PFS subgroup analysis. For OS, elevated PLR did not reach statistical significance in triple-negative breast cancer patients, while all other cancer types demonstrated a significant inverse association between high PLR and OS. The most prominent negative correlations were observed in hepatocellular carcinoma (HR = 2.10, 95% CI: 1.43-3.08), esophageal carcinoma (HR = 2.08, 95% CI: 1.13-3.83), and head and neck squamous cell carcinoma (HNSCC, HR = 2.61, 95% CI: 1.70-4.00). Significant OS reduction with high PLR was also evident in non-small cell lung cancer (NSCLC, HR = 1.80, 95% CI: 1.41-2.29) and renal cell carcinoma (HR = 1.90, 95% CI: 1.29-2.80). Regarding PFS, the predictive value of high PLR varied across cancer types. Significant associations with reduced PFS were observed in gastric cancer (HR = 1.40, 95% CI: 1.15-1.70), NSCLC (HR = 1.59, 95% CI: 1.27-1.99), hepatocellular carcinoma (HR = 1.78, 95% CI: 1.36-2.34), and esophageal carcinoma (HR = 1.67, 95% CI: 1.08-2.59). However, in subgroups such as renal cell carcinoma (HR = 1.60, 95% CI: 0.97-2.62), although high PLR suggested increased PFS risk, the results did not reach statistical significance. Overall, the negative predictive effect of high PLR on OS was more universal across cancer types receiving immunotherapy, while its impact on PFS demonstrated variability depending on specific cancer characteristics (**Supplementary Figure S1**).

#### 3.3.3 PLR cutoff value subgroup analysis

Acknowledging the heterogeneity of cutoff values in the literature and selecting PLR = 180 as a frequently reported empirical threshold, all included studies were divided into PLR≥180 (high PLR group) and PLR<180 (low PLR group) subgroups to analyze the prognostic value of PLR for OS and PFS under different thresholds. For OS, the pooled results for the PLR≥180 subgroup demonstrated that high PLR was significantly associated with shorter OS (HR = 1.87, 95% CI: 1.59-2.20). In the PLR<180 subgroup, the risk of OS reduction associated with high PLR was slightly lower (HR = 1.73, 95% CI: 1.47-2.03). The test for subgroup differences indicated no statistically significant difference in the predictive value for OS between the two cutoff subgroups (*χ²* = 0.49, df=1, p=0.48). For PFS, in the PLR≥180 subgroup, high PLR was significantly associated with reduced PFS (HR = 1.68, 95% CI: 1.43-1.98). In the PLR<180 subgroup, high PLR was associated with an increased risk of disease progression (HR = 1.53, 95% CI: 1.33-1.76) (see **Supplementary Figure S2**).

#### 3.3.4 ICI class subgroup analysis

**Figure 5:**
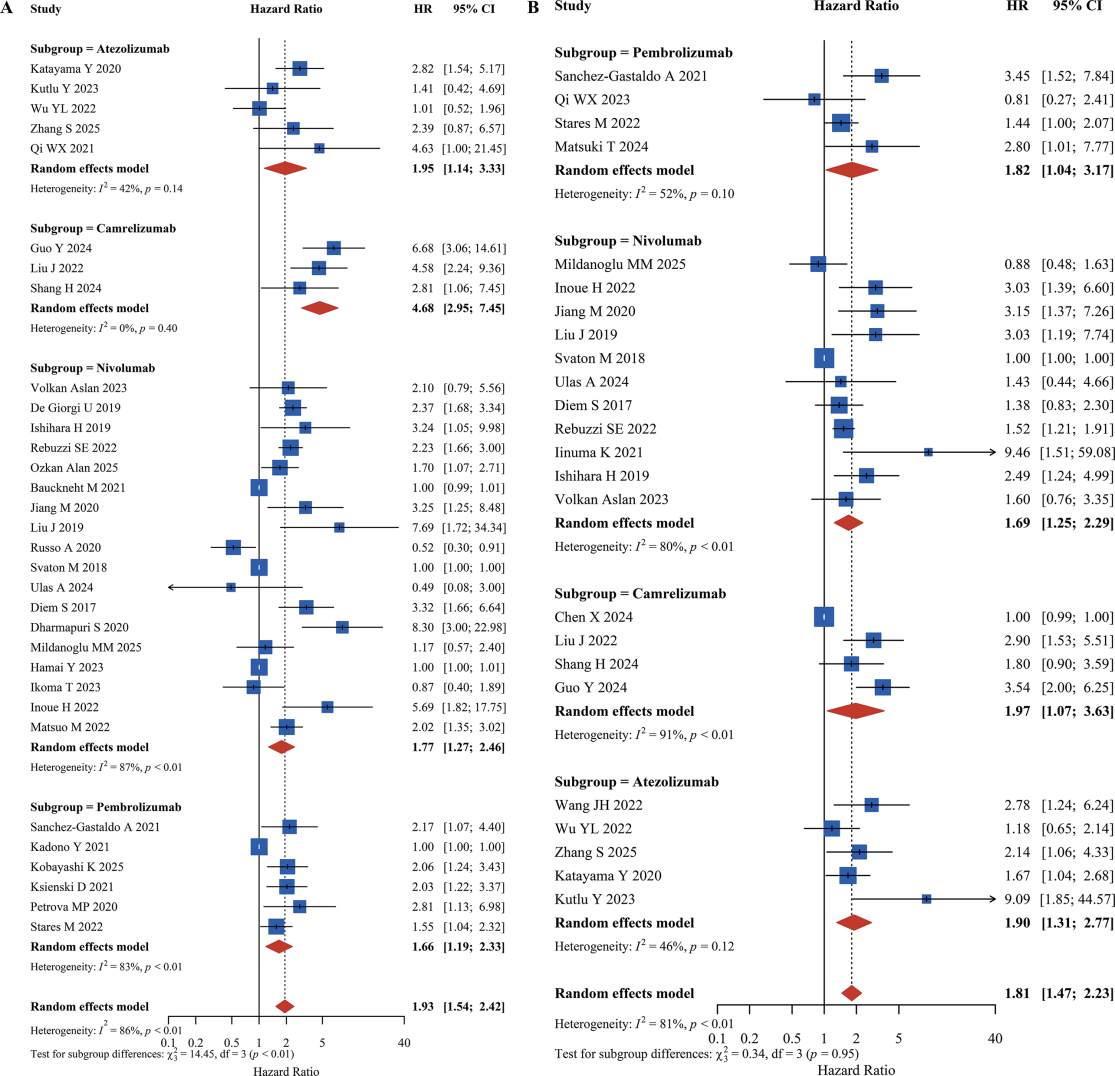
Forest plot of ICI Class subgroup analysis **(A)** Overall Survival (OS). **(B)** Progression-Free Survival (PFS).

Subgroup analyses were performed for pembrolizumab, camrelizumab, atezolizumab, and other ICIs. Results showed that high PLR posed a significant risk for both OS and PFS across different ICI treatments. For OS, the camrelizumab subgroup showed the strongest association between high PLR and OS reduction (HR = 4.68, 95% CI: 2.95-7.45). Significant OS reduction was also observed with pembrolizumab (HR = 1.66, 95% CI: 1.19-2.33), atezolizumab (HR = 1.95, 95% CI: 1.14-3.33), and nivolumab (HR = 1.77, 95% CI: 1.27-2.46). For PFS, the strength of association was relatively consistent across ICI subgroups: atezolizumab subgroup (HR = 1.90, 95% CI: 1.31-2.77), camrelizumab subgroup (HR = 1.97, 95% CI: 1.07-3.63), pembrolizumab subgroup (HR = 1.82, 95% CI: 1.04-3.17), and nivolumab subgroup (HR = 1.69, 95% CI: 1.25-2.29) all indicated that high PLR was associated with reduced PFS, with no significant difference between subgroups, as shown in **Figure 5**.

#### 3.3.5 Subgroup analysis by first-line and second-line therapy

**Figure 6:**
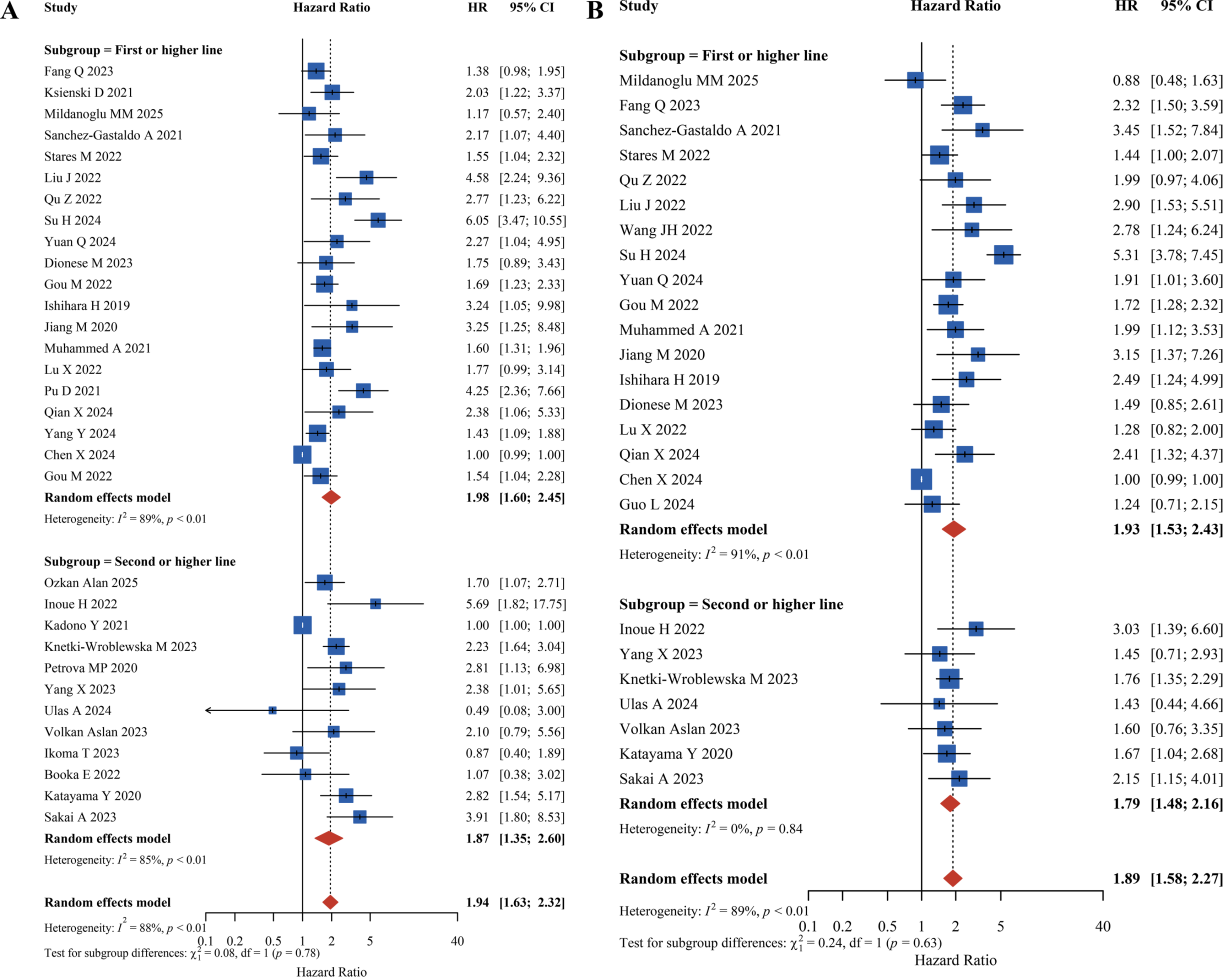
Forest plot of first-line *vs* second-line therapy subgroup analysis **(A)** Overall Survival (OS). **(B)** Progression-Free Survival (PFS).

This study categorized patients into ‘first-line or above’ and ‘second-line or above’ subgroups based on the line of treatment to analyze the prognostic impact of PLR on OS and PFS. For OS, in the first-line or above subgroup, high PLR was significantly associated with shortened OS (HR = 1.98, 95% CI: 1.60–2.45). Similarly, in the second-line or above subgroup, high PLR was significantly associated with shortened OS (HR = 1.87, 95% CI: 1.35–2.60). For PFS, the association between high PLR and reduced PFS was more pronounced in the first-line or above subgroup (HR = 1.93, 95% CI: 1.53–2.43), while it was slightly weaker but still significant in the second-line or above subgroup (HR = 1.79, 95% CI: 1.48–2.16). Overall, regardless of whether patients received first-line or above or second-line or above immunotherapy, high PLR was significantly associated with shortened OS and PFS, with no significant differences observed between the treatment line subgroups, indicating that the prognostic value of PLR is not influenced by the line of treatment, as shown in **Figure 6**.

#### 3.3.6 Tumor stage subgroup analysis

Subgroup analysis by tumor stage demonstrated that in OS-related studies, high PLR showed a stronger association with OS in stage III-IV patients (HR = 2.02, 95% CI: 1.39-2.93). Significant OS reduction was also observed in stage IIIB-IV (HR = 1.81, 95% CI: 1.08-3.03) and stage IV (HR = 1.96, 95% CI: 1.55-2.48) patients with high PLR. In liver cancer-specific staging, neither BCLC B/C stage (HR = 1.28, 95% CI: 0.90-1.82) nor BCLC C stage (HR = 1.42, 95% CI: 0.62-3.25) showed statistical significance. In PFS-related studies, high PLR was significantly associated with reduced PFS in stage IIIB-IV (HR = 1.44, 95% CI: 1.08-1.91), stage III-IV (HR = 1.71, 95% CI: 1.12-2.61), BCLC B/C stage (HR = 1.36, 95% CI: 1.07-1.73), and stage IV (HR = 1.44, 95% CI: 1.26-1.65) patients (**Supplementary Figure S3**).

### 3.4 Urological cancer subgroup analysis

This study pooled data from all included studies on urological cancer patients and analyzed the prognostic impact of PLR on OS and PFS by drug type, treatment line, and tumor stage. For OS, all subgroups indicated an association between high PLR and shortened OS. In the drug type subgroup, high PLR significantly reduced OS in patients treated with nivolumab (HR = 2.31, 95% CI: 1.86-2.86). In tumor stage subgroup analysis, high PLR significantly increased mortality risk in stage IV patients (HR = 2.33, 95% CI: 1.92-2.82), while no prognostic significance of PLR for OS was observed in patients with advanced disease without specific staging (HR = 1.53, 95% CI: 0.97-2.40).

**Figure 7:**
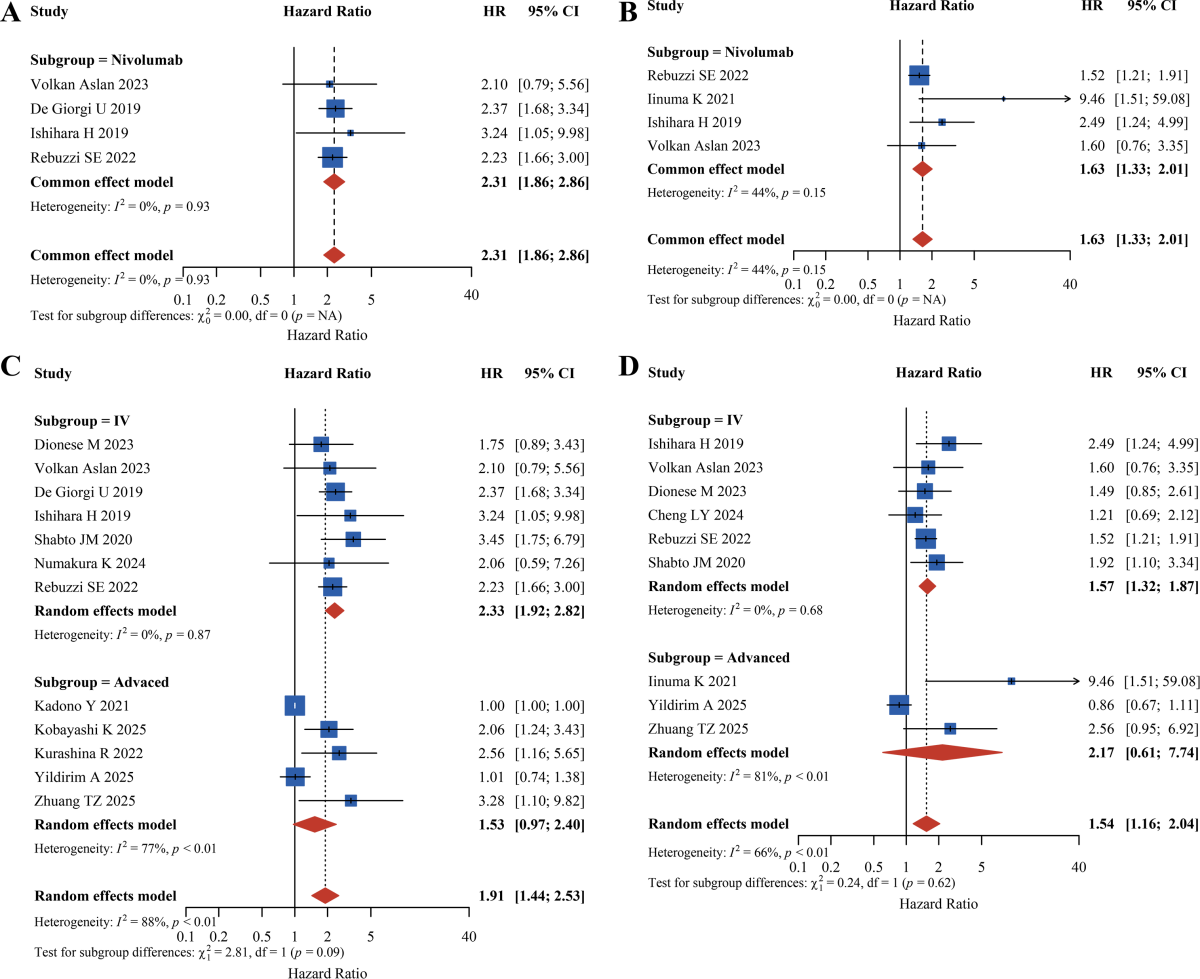
Forest plot of urological cancer subgroup analysis figure **(A)** and Figure **(C)** pertain to the analysis of Overall Survival (OS), while Figure **(B)** and Figure **(D)** pertain to the analysis of Progression-Free Survival (PFS).

For PFS, high PLR was similarly associated with reduced PFS. The drug type subgroup (nivolumab) showed a pooled HR = 1.63 (95% CI: 1.33-2.01), indicating significant negative predictive value of high PLR for PFS in nivolumab-treated patients. In the tumor stage subgroup, the association was more stable in the stage IV subgroup (HR = 1.57, 95% CI: 1.32-1.87) (**Figure 7**).

### 3.5 Non-small cell lung cancer subgroup analysis

Analysis of NSCLC patient data evaluated PLR’s prognostic impact on OS and PFS by drug class, treatment line, and tumor stage. In OS studies, PLR failed to demonstrate a clear prognostic link to OS in the nivolumab subgroup (HR = 1.47, 95% CI: 0.84-2.57), whereas elevated PLR remained a strong predictor of diminished OS in pembrolizumab-treated patients (HR = 1.86, 95% CI: 1.42-2.45). In treatment line subgroups, high PLR significantly shortened OS in both first-line or above (HR = 2.37, 95% CI: 1.68-3.34) and second-line or above subgroups (HR = 2.14, 95% CI: 1.71-2.69). However, no significant OS association was observed in stage IIIB-IV patients (HR = 1.61, 95% CI: 0.88-2.95).

Regarding PFS outcomes, PLR did not reach statistical significance in nivolumab-treated patients (HR = 1.61, 95% CI: 0.99-2.62). Both first-line and second-line or above immunotherapy subgroups showed significantly reduced PFS with high PLR. No significant PFS associations were observed in stage IIIB-IV (HR = 1.21, 95% CI: 0.92-1.58) and stage III-IV (HR = 2.25, 95% CI: 0.95-5.33) subgroups (**Supplementary Materials Figures S4**-**S9**).

### 3.6 Gastrointestinal cancer subgroup analysis

Analysis of gastrointestinal cancer data assessed PLR’s prognostic value across drug classes, treatment lines, and tumor stages. Regarding OS, elevated PLR emerged as a particularly strong determinant of poor OS in camrelizumab-treated patients (HR = 2.87, 95% CI: 1.19-6.96), yet failed to establish a clear prognostic correlation within the atezolizumab or nivolumab subgroups. High PLR significantly shortened OS in first-line or above therapy (HR = 1.60, 95% CI: 1.22-2.10), but not in second-line or above therapy (HR = 1.79, 95% CI: 0.81-3.99). PLR exhibited a profound impact on OS for patients in stage III-IV (HR = 1.60, 95% CI: 1.19-2.17) and advanced-stage (HR = 2.04, 95% CI: 1.51-2.76) categories, whereas its prognostic relevance was not statistically established for the stage BCLC B/C and stage IV subgroups.

For PFS, high PLR significantly reduced PFS in camrelizumab (HR = 1.97, 95% CI: 1.07-3.63) and atezolizumab (HR = 1.81, 95% CI: 1.08-3.04) subgroups. High PLR significantly shortened PFS in first-line or above therapy (HR = 1.60, 95% CI: 1.22-2.10) but not in second-line or above therapy. Significant PFS reduction was observed in stage III-IV (HR = 1.46, 95% CI: 1.08-1.98), stage BCLC C(HR = 1.81, 95% CI: 1.08-3.04) and advanced-stage (HR = 1.93, 95% CI: 1.38-2.71) subgroups, while no significant associations were found in BCLC B/C and stage IV subgroups (**Supplementary Materials S10**-**S15**).

### 3.7 Nivolumab subgroup analysis by cancer type

Analysis of nivolumab-treated patients evaluated the prognostic utility of PLR across cancer types. For OS, high PLR lacked a clear correlation with OS in the ESCC (HR = 1.54, 95% CI: 0.53-4.41) or NSCLC (HR = 1.47, 95% CI: 0.84-2.57) subgroups, but was robustly linked to shortened OS in RCC (HR = 2.31, 95% CI: 1.86-2.86). The prognostic relevance for OS remained inconclusive for other cancer types (HR = 2.53, 95% CI: 0.88-7.28).

For PFS, high PLR failed to reach statistical significance in NSCLC (HR = 1.61, 95% CI: 0.99-2.62) but consistently predicted poorer PFS in RCC (HR = 1.79, 95% CI: 1.27-2.51). (**Supplementary Materials Figures S16**, **S17**).

### 3.8 Meta-regression analysis

Multivariable meta-regression was conducted to explore potential sources of heterogeneity by simultaneously adjusting for cancer type, therapy line, disease stage, and other covariates. For OS, the multivariable model accounted for 32.84% of the between-study variance (*P* = 0.0045). After adjustment, cancer type (HCC, *P* = 0.004; HNSCC, *P* = 0.013), therapy line (second or higher, *P* = 0.039), and disease stage (Stage III–IV, *P* = 0.019) remained significant independent predictors of heterogeneity.

For PFS, the model explained 24.62% of heterogeneity (*P* = 0.0504). Therapy line (*P* = 0.008) and disease stage (*P* = 0.020) were identified as significant independent moderators, whereas cancer type and ICI class did not show statistical significance in the adjusted model. These findings suggest that treatment line and disease stage are robust determinants of the prognostic heterogeneity, independent of other clinical characteristics. (**Supplementary Materials Tables S3**, **S4**).

### 3.9 Publication bias assessment

**Figure 8:**
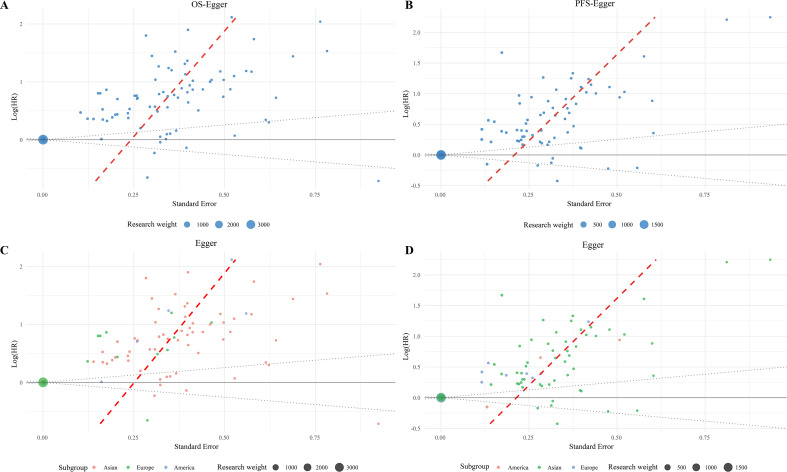
Scatter plots for bias analysis [**(A)** Egger’s test using OS data; **(B)** Egger’s test using PFS data; **(C)** Egger’s test using OS data with geographic subgroups; **(D)** Egger’s test using PFS data with geographic subgroups].

Funnel plots and Egger’s/Begg’s tests assessed publication bias for OS and PFS outcomes. For OS, Egger’s test indicated significant bias (*P* < 0.05) while Begg’s test showed no significant bias (*P* = 0.926). For PFS, Egger’s test also indicated significant bias (*P* < 0.05) while Begg’s test showed no significant bias (*P* = 0.655). Asymmetric funnel plot distributions suggested potential publication bias. Given these findings, Duval and Tweedie’s non-parametric trim-and-fill method was applied to further evaluate the robustness of the pooled results. For OS, the analysis identified 40 potentially missing studies on the left side of the funnel plot. After imputing these studies, the adjusted pooled HR shifted to 1.105 (95% CI: 0.939–1.300, *P* = 0.231). Similarly, for PFS, 36 missing studies were imputed, resulting in an adjusted HR of 1.004 (95% CI: 0.993–1.016, *P* = 0.467). These results suggest that the original prognostic estimates for both OS and PFS may have been overestimated due to publication bias (**Figure 8**; **Supplementary Figures S18**-**S21**, **Supplementary Table S5**).

### 3.10 Sensitivity analysis

To assess the robustness of our findings, sensitivity analyses were performed by stratifying studies according to their Newcastle–Ottawa Scale (NOS) scores. For OS, the pooled HR remained significant in the high-quality subgroup (NOS ≥ 9; 26 studies: HR 1.06, 95% CI 1.03–1.10, *P* = 0.0003), while no significant association was observed in lower-quality studies (NOS < 9; 60 studies: HR 1.0, 95% CI 1.0–1.01, *P* = 0.18). Similarly, for PFS, high-quality studies (n = 26) yielded a significant HR of 1.06 (95% CI 1.03–1.10, *P* = 0.0001), whereas the association in lower-quality studies (n = 46) was not statistically significant (HR 1.01, 95% CI 1.0–1.01, *P* = 0.2452). These results demonstrate that the primary conclusions are predominantly driven by high-quality evidence, confirming the stability and reliability of the overall estimates. (**Figure 8**; **Supplementary Table S6**, **Supplementary Figure S22**).

## 4 Discussion

The advent of cancer immunotherapy has brought revolutionary breakthroughs in the treatment of solid tumors. Immune checkpoint inhibitors, particularly PD-1/PD-L1 inhibitors, have demonstrated survival benefits across multiple malignancies including NSCLC, renal cell carcinoma, and melanoma. For instance, the 5-year survival rate for advanced NSCLC patients receiving ICIs has increased to 15%-20%, far surpassing the <5% rate observed in the chemotherapy era (116). However, clinical practice continues to face two major challenges. First, the efficacy of ICIs demonstrates significant heterogeneity, with only 20%-40% of patients achieving sustained responses. The remaining patients may experience treatment discontinuation due to primary resistance, an immunosuppressive tumor microenvironment, or immune-related adverse events (irAEs) (117). Second, PD-L1 expression testing is hampered by issues such as differences in antibody clones and false negatives caused by tumor heterogeneity. Tumor mutational burden (TMB) testing is characterized by high costs and long turnaround times, limiting its widespread adoption in primary care settings. Meanwhile, microsatellite instability-high (MSI-H) or mismatch repair deficiency (dMMR) is predominantly observed in only a few cancer types, such as colorectal and endometrial cancers, and cannot fulfill the need for pan-cancer prognostic assessment (118–120). Consequently, there is an urgent need to identify novel biomarkers. The PLR, as a peripheral blood marker reflecting systemic inflammatory and immune balance, represents a promising direction for further investigation.

The prognostic value of PLR is hypothesized to reflect the complex interplay between systemic inflammation and tumor immunity. Although direct mechanistic links were not evaluated in the included clinical studies, we speculate that activated platelets might release cytokines such as platelet-derived growth factor (PDGF) and transforming growth factor-β (TGF-β). These factors could theoretically facilitate tumor angiogenesis and potentially foster an immunosuppressive microenvironment by recruiting myeloid-derived suppressor cells (MDSCs) and regulatory T cells (Tregs), which might inhibit the function of CD8^+^ cytotoxic T cells (121, 122). Concurrently, an elevated PLR is often accompanied by reduced lymphocyte counts, which can directly determine the intensity of the immune response (123). Previous single-center studies have preliminarily confirmed the association between high PLR and poor outcomes following ICI therapy. For instance, a study by Xu et al. showed that patients with PLR ≥170 had a 102% increased risk of shorter median OS compared to those with PLR <170 (HR = 2.02, 95% CI: 1.46 to 2.80), and a 74% increased risk of shorter PFS (HR = 1.74, 95% CI: 1.27-2.38) (124). However, these studies were mostly limited to single cancer types or single ICI classes, and the lack of a unified PLR cutoff value hindered cross-study comparisons and the ability to define its prognostic value across different treatment lines and tumor stages (125, 126). Furthermore, systematic evidence is still lacking regarding the differential prognostic role of PLR for specific ICIs (e.g., nivolumab) across various cancer types, as well as its synergistic predictive power combined with other inflammatory markers, which significantly limits the clinical translation of PLR.

This systematic review and meta-analysis, encompassing 98 studies and patient data across 10 cancer types, aimed to address these research gaps. The results demonstrated that elevated PLR consistently served as a risk factor for OS in various malignancies, including NSCLC, gastrointestinal cancers, urological cancers, and renal cell carcinoma, significantly shortening OS. The association was most pronounced in esophageal carcinoma (HR = 2.08, 95% CI: 1.13-3.83) and hepatocellular carcinoma (HR = 2.10, 95% CI: 1.43-3.08), a finding consistent with the chronic inflammatory microenvironment characteristic of these cancers (127). Esophageal carcinoma patients often present with long-standing esophageal mucosal inflammation, while hepatocellular carcinoma patients frequently have underlying viral hepatitis or cirrhosis, resulting in a high baseline inflammatory burden. Elevated PLR, as a manifestation of systemic inflammation, may reflect an immunosuppressive microenvironment that undermines ICI efficacy (128–130). Notably, no statistically significant difference was reached in the OS subgroup for triple-negative breast cancer, potentially related to the immunogenic heterogeneity of TNBC. Some TNBC patients harbor BRCA mutations or high TMB, whose robust immune responses might counteract the negative impact of high PLR (131–133).

Regarding progression-free survival, the prognostic value of elevated PLR demonstrated significant cancer-type specificity: high PLR was significantly associated with shortened PFS in NSCLC (HR = 1.59, 95% CI: 1.27-1.99), hepatocellular carcinoma (HR = 1.78, 95% CI: 1.36-2.34), and esophageal carcinoma (HR = 1.67, 95% CI: 1.08-2.59). However, this association did not reach statistical significance in renal cell carcinoma (HR = 1.60, 95% CI: 0.97-2.62). This discrepancy may be attributed to differences in cancer biology. RCC patients receiving ICI therapy are prone to pseudoprogression, potentially introducing assessment bias in radiological PFS evaluation (134, 135). Furthermore, the non-significant PFS result in the main RCC analysis contrasted with the robust correlation observed in the nivolumab-treated RCC subgroup. This divergence likely stems from the masking effect of pharmacological heterogeneity; the inclusion of various ICIs in the main analysis may have diluted the specific prognostic signal. In contrast, the more homogenous nivolumab-RCC subgroup unmasked the potent prognostic utility of PLR for this specific agent. In TNBC patients, fluctuations in lymphocyte counts due to chemotherapy-induced myelosuppression may obscure the relationship between PLR and PFS, necessitating further investigation (136, 137).

Subgroup analysis by ICI type further revealed potential mechanistic interactions between PLR and different inhibitors. Among patients receiving camrelizumab, high PLR demonstrated the most pronounced effect on OS reduction (HR = 4.68, 95% CI: 2.95-7.45), whereas the associations were more moderate in nivolumab and pembrolizumab subgroups (HR = 1.77 and 1.66, respectively). We speculate that this discrepancy might be partially related to camrelizumab’s structural features, such as its Fc segment properties. It is hypothesized that in patients with high PLR, the elevated systemic inflammatory burden might interfere with the immune-modulatory effects of specific antibodies, although this requires further biological validation (138, 139). In contrast, nivolumab and pembrolizumab primarily modulate the immune microenvironment through direct CD8^+^ T-cell activation, and high PLR might exert a general inhibitory effect on this process, resulting in more consistent PFS outcomes across ICI subgroups (140). Notably, nivolumab demonstrated unique prognostic value for PLR in the RCC subgroup (OS: HR = 2.31, 95% CI: 1.86-2.86; PFS: HR = 1.63, 95% CI: 1.33-2.01), but not in NSCLC or esophageal carcinoma subgroups. This may be hypothetically linked to RCC’s characteristic VEGF overexpression (141). It is suggested that platelet-derived factors might contribute to angiogenesis, potentially creating barriers to immune cell infiltration. Phase III clinical trials have confirmed that nivolumab combined with anti-VEGF agents can disrupt this cycle and improve RCC outcomes, suggesting that RCC patients with high PLR may be more suitable for ICI plus anti-VEGF combination therapy (142, 143). Beyond single inflammatory indices, composite markers like the C-PLAN index (comprising CRP, PLR, Albumin, and NLR) have recently demonstrated superior prognostic accuracy in nivolumab-treated RCC by integrating multiple systemic pathways. While PLR specifically reflects the balance between thrombocytosis-driven tumor promotion and lymphocytic immune surveillance, composite scores like C-PLAN may offer incremental value by incorporating nutritional status and acute-phase reactants, providing a more comprehensive reflection of the tumor-host interface in RCC (144).

Subgroup analyses by treatment line and tumor stage further expand the clinical applicability of PLR. Elevated PLR was consistently associated with reduced OS/PFS regardless of whether patients received first-line or second-line (and beyond) ICI therapy, though some outcomes in the second-line subgroup (e.g., OS in urological cancers) did not reach statistical significance. This may be attributed to poorer baseline performance status and confounding effects from prior radiotherapy or chemotherapy in later-line patients. These findings suggest that patients with high pre-treatment PLR in the first-line setting may harbor primary immune resistance risks, warranting consideration for initial ICI-chemotherapy combinations to enhance efficacy. For second-line therapy, elevated PLR reflects inflammation-immune imbalance exacerbated by previous treatments, necessitating shorter radiological assessment intervals for early progression detection. In tumor stage subgroup analysis, the prognostic impact of high PLR was more pronounced in stage IV patients, while earlier-stage subgroups (e.g., II-IV) showed relatively modest effects. This discrepancy may stem from advanced cancer patients frequently exhibiting more severe systemic inflammatory responses and pre-cachectic states, where PLR as an integrative inflammatory marker better reflects overall immune suppression. Conversely, early-stage patients with lower tumor burden experience less tumor-driven inflammation, diminishing PLR’s predictive weight. This underscores the need for dynamic interpretation of PLR values in context with tumor staging. Furthermore, our analysis revealed similar hazard ratios for the subgroups defined by different PLR cutoffs (e.g., <180 *vs*. ≥180). This similarity suggests that PLR likely functions as a continuous prognostic risk factor rather than exhibiting a distinct “cliff effect” at a specific numerical threshold. Consequently, the clinical implication is that a progressive elevation in PLR signals a generally worsening prognosis; therefore, clinical decision-making should consider the overall trend of inflammatory burden rather than relying solely on a single rigid cutoff point.

## 5 Limitations

This study has several limitations: (1) The included studies were predominantly retrospective in design, introducing potential selection bias; variations in the completeness of records regarding patient comorbidities and prior treatment history may affect the stability of the results. (2) The lack of a standardized cutoff value for PLR, with differing risk stratification thresholds across studies, may lead to bias in effect size calculations and weaken cross-study comparability. (3) Therapeutic coverage for certain malignancies is constrained by data availability; for instance, while RCC has numerous frontline options, eligible studies reporting PLR-stratified HRs were largely restricted to nivolumab-based regimens. This scarcity of data on newer ICI-TKI combinations may limit the generalizability of PLR’s prognostic utility across the entire current RCC treatment landscape. (4) A critical limitation is that, as the included primary studies predominantly reported results in the form of categorical variables (high *vs*. low), we were unable to access raw data to conduct analyses treating PLR as a continuous variable. This constraint inevitably leads to a loss of information regarding the linear dose-response relationship and prevents the establishment of a precise prognostic nomogram. (5) Due to the lack of direct biological measurements in the included studies, the mechanistic explanations discussed herein (e.g., regarding specific cytokines or cellular interactions) remain speculative and hypothesis-generating. Future translational research is needed to causally validate these pathways. (6) Only the prognostic role of baseline PLR was explored, without incorporating dynamic changes in PLR during treatment. Based on these limitations, future research should focus on three key directions: (1) Conducting multicenter prospective cohort studies with standardized PLR measurement timing and cutoff values. (2) Designing longitudinal study protocols to analyze the association between the magnitude of PLR reduction after 2–4 treatment cycles and ICI efficacy. (3) Integrating basic science experiments with clinical samples to investigate the molecular mechanisms through which PLR modulates the tumor microenvironment.

## 6 Conclusion

This study demonstrates that elevated baseline platelet-to-lymphocyte ratio is associated with poor prognosis in cancer patients receiving immune checkpoint inhibitors, manifested as significantly shortened overall survival across most cancer types and reduced progression-free survival in specific malignancies including non-small cell lung cancer, hepatocellular carcinoma, and esophageal carcinoma. This prognostic value is particularly prominent in renal cell carcinoma patients treated with nivolumab, advanced gastrointestinal cancer patients, and those receiving first-line ICI therapy. As an inexpensive and readily accessible peripheral blood marker, PLR holds clinical significance for initial prognostic stratification in ICI-treated patients. However, multicenter randomized controlled trials are warranted to establish optimal PLR cutoff values for different cancer types, validate the prognostic significance of dynamic PLR changes, and further explore its prognostic implications across various malignancies.

## Data Availability

The original contributions presented in the study are included in the article/**Supplementary Material**. Further inquiries can be directed to the corresponding author.

## Supplementary material

Supplementary Table 1 Search Strategy.

Supplementary Table 2 Quality Assessment.

Supplementary Table 3 Meta-regression for OS.

Supplementary Table 4 Meta-regression for PFS.

Supplementary Table 5 Sensitivity Analysis of Publication Bias Using the Trim-and-Fill Method for OS and PFS.

Supplementary Table 6 Sensitivity Analysis for OS and PFS Stratified by Study Quality Based on NOS Scores.

Supplementary Figure 1 Forest Plots of Cancer Type Subgroup Analysis.

Supplementary Figure 2 Forest Plots of PLR Cutoff Value Subgroup Analysis.

Supplementary Figure 3 Forest Plots of Tumor Stage Subgroup Analysis.

Supplementary Figure 4 Forest Plots of NSCLC Drug Subgroup Analysis (OS).

Supplementary Figure 5 Forest Plots of NSCLC Treatment Line Subgroup Analysis (OS).

Supplementary Figure 6 Forest Plots of NSCLC Stage Subgroup Analysis (OS).

Supplementary Figure 7 Forest Plots of NSCLC Drug Subgroup Analysis (PFS).

Supplementary Figure 8 Forest Plots of NSCLC Treatment Line Subgroup Analysis (PFS).

Supplementary Figure 9 Forest Plots of NSCLC Stage Subgroup Analysis (PFS).

Supplementary Figure 10 Forest Plots of Gastrointestinal Cancer Drug Subgroup Analysis (OS).

Supplementary Figure 11 Forest Plots of Gastrointestinal Cancer Treatment Line Subgroup Analysis (OS).

Supplementary Figure 12 Forest Plots of Gastrointestinal Cancer Stage Subgroup Analysis (OS).

Supplementary Figure 13 Forest Plots of Gastrointestinal Cancer Drug Subgroup Analysis (PFS).

Supplementary Figure 14 Forest Plots of Gastrointestinal Cancer Treatment Line Subgroup Analysis (PFS).

Supplementary Figure 15 Forest Plots of Gastrointestinal Cancer Stage Subgroup Analysis (PFS).

Supplementary Figure 16 Forest Plots of Cancer Type Subgroup Analysis in Nivolumab-Treated Patients (OS).

Supplementary Figure 17 Forest Plots of Cancer Type Subgroup Analysis in Nivolumab-Treated Patients (PFS).

Supplementary Figure 18 Funnel Plot of OS Data.

Supplementary Figure 19 Funnel Plot of PFS Data.

Supplementary Figure 20 Funnel plot with trim-and-fill analysis for OS.

Supplementary Figure 21 Funnel plot with trim-and-fill analysis for PFS.

Supplementary Figure 22 Forest plots of sensitivity analyses for (A) OS and (B) PFS stratified by study quality.
