# Supplementary material for: Platelet-to-lymphocyte ratio for prognostication in immune checkpoint inhibitor-treated cancer patients: a meta-analysis of 13027 patients highlighting nivolumab-responsive renal cell carcinoma
Source: Front Immunol. 2026 Feb 2;17:1732790. doi: 10.3389/fimmu.2026.1732790 (PMC12907331; doi:10.3389/fimmu.2026.1732790)
Supplement: Supplementary Table 3 — Meta-regression for OS. [file Table3.docx]

**Identification of studies via databases and registers**

Records identified from*:

PubMed (n=250),

Embase (n=544),

Cochrane Library (n=69),

Web of Science (n=377)

Records removed *before screening*:

Duplicate records removed (n =292)

**Identification**

Records excluded:

Meta analysis (n=42)

Review (n=10)

Records screened

(n =948)

Records excluded:

Differing study outcomes (=172)

Pharmacological experiments (n=9)

Animal experiments (n=10)

Others (n=28)

Records screened

(n = 896)

**Screening**

Reports excluded:

Subject mismatch (n=347)

Clinical indicators do not match (n=58)

Insufficient data (n=174)

Reports assessed for eligibility

(n = 677)

Studies included in review

(n =98)

**Included**

*Consider, if feasible to do so, reporting the number of records identified from each database or register searched (rather than the total number across all databases/registers).

**If automation tools were used, indicate how many records were excluded by a human and how many were excluded by automation tools.

*From:*  Page MJ, McKenzie JE, Bossuyt PM, Boutron I, Hoffmann TC, Mulrow CD, et al. The PRISMA 2020 statement: an updated guideline for reporting systematic reviews. BMJ 2021;372:n71. doi: 10.1136/bmj.n71

For more information, visit: <http://www.prisma-statement.org/>
