# Supplementary material for: Platelet-to-lymphocyte ratio for prognostication in immune checkpoint inhibitor-treated cancer patients: a meta-analysis of 13027 patients highlighting nivolumab-responsive renal cell carcinoma
Source: Front Immunol. 2026 Feb 2;17:1732790. doi: 10.3389/fimmu.2026.1732790 (PMC12907331; doi:10.3389/fimmu.2026.1732790)
Supplement: Supplementary Table 4 — Meta-regression for PFS. [file Table4.docx]

Supplementary Materials

## Table S1. Detailed Search Strategy

| Pubmed: 250 | (((((((((((((((((((((((((((((((Immune Checkpoint Inhibitors[MeSH Terms]) OR (Checkpoint Inhibitors, Immune[Title/Abstract])) OR (Immune Checkpoint Blockers[Title/Abstract])) OR (Immune Checkpoint Inhibitor[Title/Abstract])) OR (Checkpoint Inhibitor, Immune[Title/Abstract])) OR (CTLA-4 Inhibitors[Title/Abstract])) OR (CTLA 4 Inhibitors[Title/Abstract])) OR (Cytotoxic T-Lymphocyte-Associated Protein 4 Inhibitors[Title/Abstract])) OR (CTLA-4 Inhibitor[Title/Abstract])) OR (PD-1 Inhibitors[Title/Abstract])) OR (Programmed Cell Death Protein 1 Inhibitor[Title/Abstract])) OR (Inhibitor, PD-1[Title/Abstract])) OR (Immune Checkpoint Blockade[Title/Abstract])) OR (Checkpoint Inhibition, Immune[Title/Abstract])) OR (PD-L1 Inhibitors[Title/Abstract])) OR (Programmed Death-Ligand 1 Inhibitors[Title/Abstract])) OR (PD-L1 Inhibitor[Title/Abstract])) OR (PD-1-PD-L1 Blockade[Title/Abstract])) OR (PD 1 PD L1 Blockade[Title/Abstract])) OR (nivolumab[Title/Abstract])) OR (pembrolizumab[Title/Abstract])) OR (camrelizumab[Title/Abstract])) OR (sintilimab[Title/Abstract])) OR (toripalimab[Title/Abstract])) OR (atezolizumab[Title/Abstract])) OR (durvalumab[Title/Abstract])) OR (avelumab[Title/Abstract])) OR (sugemalimab[Title/Abstract])) OR (envafolimab[Title/Abstract])) AND (((((((thrombocyte[MeSH Terms]) ) OR (blood platelet[Title/Abstract])) OR (platelets[Title/Abstract])) OR (platelet[Title/Abstract])) OR (thrombocytes[Title/Abstract])) OR (thrombocyte[Title/Abstract]))) AND ((((lymphocyte[MeSH Terms]) OR (Lymphocyte[Title/Abstract])) OR (Lymphoid Cells[Title/Abstract])) OR (Lymphoid Cell[Title/Abstract]))) AND ((((((((((((((((((Neoplasms[MeSH Terms]) OR (Tumors[Title/Abstract])) OR (Neoplasia[Title/Abstract])) OR (Neoplasias[Title/Abstract])) OR (Neoplasm[Title/Abstract])) OR (Tumor[Title/Abstract])) OR (Cancer[Title/Abstract])) OR (Cancers[Title/Abstract])) OR (Malignant Neoplasm[Title/Abstract])) OR (Malignancy[Title/Abstract])) OR (Malignancies[Title/Abstract])) OR (Malignant Neoplasms[Title/Abstract])) OR (Neoplasm, Malignant[Title/Abstract])) OR (Neoplasms, Malignant[Title/Abstract])) OR (Benign Neoplasms[Title/Abstract])) OR (Neoplasms, Benign[Title/Abstract])) OR (Neoplasm, Benign[Title/Abstract])) OR (Benign Neoplasm[Title/Abstract])) |
| --- | --- |
| Embase: 544 | 1#: 'immune checkpoint inhibitors'/exp OR 'immune checkpoint inhibitors' OR (('immune' OR 'immune'/exp OR immune) AND ('checkpoint' OR 'checkpoint'/exp OR checkpoint) AND ('inhibitors' OR 'inhibitors'/exp OR inhibitors)) OR 'checkpoint inhibitors, immune':ti,ab,kw OR 'immune checkpoint blockers':ab,ti OR 'checkpoint blockers, immune':ti,ab,kw OR 'immune checkpoint inhibitor':ti,ab,kw OR 'ctla-4 inhibitors':ti,ab,kw OR 'cytotoxic t lymphocyte associated protein 4':ti,ab,kw OR 'pd-1 inhibitors':ti,ab,kw OR 'immune checkpoint blockade':ti,ab,kw OR 'pd-l1 inhibitors':ti,ab,kw OR 'blockade, pd-1-pd-l1':ti,ab,kw OR nivolumab:ti,ab,kw OR 'pembrolizumab':ti,ab,kw OR camrelizumab:ti,ab,kw OR sintilimab:ti,ab,kw OR toripalimab:ti,ab,kw OR atezolizumab:ti,ab,kw OR durvalumab:ti,ab,kw OR avelumab:ti,ab,kw OR sugemalimab:ti,ab,kw OR envafolimab:ti,ab,kw  2#: 'thrombocyte'/exp OR 'thrombocyte' OR 'blood platelet':ti,ab,kw OR platelets:ti,ab,kw OR platelet:ti,ab,kw OR thrombocytes:ti,ab,kw OR thrombocyte:ti,ab,kw  3#: 'lymphocyte'/exp OR 'lymphocyte' OR lymphocyte:ti,ab,kw OR 'lymphoid cells':ti,ab,kw OR 'lymphoid cell':ti,ab,kw  4#: 'neoplasms'/exp OR neoplasms OR tumors:ti,ab,kw OR neoplasia:ti,ab,kw OR neoplasias:ti,ab,kw OR neoplasm:ti,ab,kw OR tumor:ti,ab,kw OR cancer:ti,ab,kw OR cancers:ti,ab,kw OR 'malignant neoplasm':ti,ab,kw OR malignancy:ti,ab,kw OR malignancies:ti,ab,kw OR 'malignant neoplasms':ti,ab,kw OR 'neoplasm, malignant':ti,ab,kw OR 'neoplasms, malignant':ti,ab,kw OR 'benign neoplasms':ti,ab,kw OR 'neoplasms, benign':ti,ab,kw OR 'neoplasm, benign':ti,ab,kw OR 'benign neoplasm':ti,ab,kw  5#: 'ratio'/exp OR ratio  1# AND 2# AND 3# AND 4# AND 5# |
| Cochrane of library: 69 | 1#: MeSH descriptor: [Lymphocytes] explode all trees OR (Lymphocyte):ti,ab,kw or (Lymphoid Cells):ti,ab,kw or (Lymphoid Cell):ti,ab,kw  2#: MeSH descriptor: [Immune Checkpoint Inhibitors] explode all trees OR Cytotoxic T Lymphocyte Associated Protein 4 Inhibitor OR Cytotoxic T Lymphocyte Associated Protein 4 Inhibitors OR CTLA 4 Inhibitors OR CTLA 4 Inhibitor OR CTLA 4 Inhibitor OR Cytotoxic T Lymphocyte Associated Protein 4 Inhibitors OR CTLA 4 Inhibitors OR Cytotoxic T Lymphocyte Associated Protein 4 Inhibitor OR Programmed Cell Death Protein 1 Inhibitors OR PD 1 Inhibitor OR Inhibitor, PD 1 OR PD 1 Inhibitors OR PD 1 Inhibitors OR Programmed Cell Death Protein 1 Inhibitor OR PD 1 Inhibitor OR Checkpoint Inhibitors, Immune OR Immune Checkpoint Inhibitor OR Checkpoint Inhibitor, Immune OR Immune Checkpoint Blockers OR Checkpoint Blockers, Immune OR PD 1 PD L1 Blockade OR Blockade, PD 1 PD L1 OR PD 1 PD L1 Blockade OR Immune Checkpoint Blockade OR Checkpoint Blockade, Immune OR Immune Checkpoint Inhibition OR Checkpoint Inhibition, Immune OR PD L1 Inhibitors OR PD L1 Inhibitors OR Programmed Death Ligand 1 Inhibitors OR PD L1 Inhibitor OR Programmed Death Ligand 1 Inhibitors OR PD L1 Inhibitor OR nivolumab OR pembrolizumab OR camrelizumab OR sintilimab OR toripalimab OR atezolizumab OR durvalumab OR avelumab OR sugemalimab OR envafolimab  3#: MeSH descriptor: [Neoplasms] explode all trees OR Neoplasm, Benign OR Benign Neoplasm OR Benign Neoplasms OR Neoplasms, Benign OR Neoplasias OR Neoplasm OR Tumor OR Tumors OR Neoplasia OR Cancers OR Malignancy OR Cancer OR Neoplasm, Malignant OR Malignant Neoplasms OR Malignancies OR Neoplasms, Malignant OR Malignant Neoplasm  4#: MeSH descriptor: [Blood Platelets] explode all trees OR Thrombocyte OR Platelet OR Platelets OR Blood Platelet OR Platelet, Blood OR Platelets, Blood OR Thrombocytes  1# AND 2# AND 3# AND 4# |
| Web of science: 377 | 1#: Lymphocytes (Topic) or Lymphocyte OR Lymphoid Cells OR Lymphoid Cell (Topic)  2#: Immune Checkpoint Inhibitors OR Cytotoxic T Lymphocyte Associated Protein 4 Inhibitor OR Cytotoxic T Lymphocyte Associated Protein 4 Inhibitors OR CTLA 4 Inhibitors OR CTLA 4 Inhibitor OR CTLA 4 Inhibitor OR Cytotoxic T Lymphocyte Associated Protein 4 Inhibitors OR CTLA 4 Inhibitors OR Cytotoxic T Lymphocyte Associated Protein 4 Inhibitor OR Programmed Cell Death Protein 1 Inhibitors OR PD 1 Inhibitor OR Inhibitor, PD 1 OR PD 1 Inhibitors OR PD 1 Inhibitors OR Programmed Cell Death Protein 1 Inhibitor OR PD 1 Inhibitor OR Checkpoint Inhibitors, Immune OR Immune Checkpoint Inhibitor OR Checkpoint Inhibitor, Immune OR Immune Checkpoint Blockers OR Checkpoint Blockers, Immune OR PD 1 PD L1 Blockade OR Blockade, PD 1 PD L1 OR PD 1 PD L1 Blockade OR Immune Checkpoint Blockade OR Checkpoint Blockade, Immune OR Immune Checkpoint Inhibition OR Checkpoint Inhibition, Immune OR PD L1 Inhibitors OR PD L1 Inhibitors OR Programmed Death Ligand 1 Inhibitors OR PD L1 Inhibitor OR Programmed Death Ligand 1 Inhibitors OR PD L1 Inhibitor OR nivolumab OR pembrolizumab OR camrelizumab OR sintilimab OR toripalimab OR atezolizumab OR durvalumab OR avelumab OR sugemalimab OR envafolimab  3#: Neoplasms OR Neoplasm, Benign OR Benign Neoplasm OR Benign Neoplasms OR Neoplasms, Benign OR Neoplasias OR Neoplasm OR Tumor OR Tumors OR Neoplasia OR Cancers OR Malignancy OR Cancer OR Neoplasm, Malignant OR Malignant Neoplasms OR Malignancies OR Neoplasms, Malignant OR Malignant Neoplasm  4#: Blood Platelets OR Thrombocyte OR Platelet OR Platelets OR Blood Platelet OR Platelet, Blood OR Platelets, Blood OR Thrombocytes  1# AND 2# AND 3# AND 4# |

## Table S2. Quality Assessment (Newcastle-Ottawa Scale)

| First Author | Year | Selection | | | Comparability | | Outcome | | | Score |
| --- | --- | --- | --- | --- | --- | --- | --- | --- | --- | --- |
|  |  | A | B | C | D | E | F | G | H |  |
| Dong Hyun Kim | 2024 | ★ | ★ | ★ | ★ | ★★ | ★ | ★ | ★ | 9 |
| Ozkan Alan | 2025 | ★ | ★ | ★ | ★ | ★★ | ★ | ★ | ★ | 9 |
| Volkan Aslan | 2023 | ★ | ★ | ★ |  | ★★ | ★ | ★ |  | 7 |
| Bai R | 2021 | ★ | ★ | ★ |  | ★★ | ★ | ★ | ★ | 8 |
| Bauckneht M | 2021 | ★ | ★ | ★ | ★ | ★★ | ★ | ★ |  | 8 |
| Mehmet A Bilen | 2018 | ★ | ★ | ★ | ★ | ★★ | ★ | ★ | ★ | 9 |
| Jiaxin Cao | 2023 | ★ | ★ | ★ | ★ | ★★ | ★ | ★ |  | 8 |
| Booka E | 2022 | ★ | ★ | ★ | ★ | ★★ | ★ | ★ |  | 8 |
| Chen Q | 2024 | ★ | ★ | ★ |  | ★★ | ★ | ★ |  | 7 |
| Chen X | 2024 | ★ | ★ | ★ |  | ★★ | ★ | ★ | ★ | 8 |
| Chen Y | 2021 | ★ | ★ | ★ | ★ | ★★ | ★ | ★ | ★ | 9 |
| Cheng LY | 2024 | ★ | ★ | ★ | ★ | ★★ | ★ | ★ |  | 8 |
| Da L | 2023 | ★ | ★ | ★ | ★ | ★★ | ★ | ★ | ★ | 9 |
| De Giorgi U | 2019 | ★ | ★ | ★ | ★ | ★★ | ★ | ★ |  | 8 |
| Dharmapuri S | 2020 | ★ | ★ | ★ |  | ★★ | ★ | ★ | ★ | 8 |
| Dionese M | 2023 | ★ | ★ | ★ |  | ★★ | ★ | ★ |  | 7 |
| Dong Q | 2024 | ★ | ★ | ★ | ★ | ★★ | ★ | ★ | ★ | 9 |
| Fan X | 2021 | ★ | ★ | ★ | ★ | ★★ | ★ | ★ | ★ | 9 |
| Fang Q | 2023 | ★ | ★ | ★ | ★ | ★★ | ★ | ★ |  | 8 |
| Gou M | 2022 | ★ | ★ | ★ | ★ | ★★ | ★ | ★ |  | 8 |
| Gou M | 2022 | ★ | ★ | ★ | ★ | ★★ | ★ | ★ |  | 8 |
| Guo L | 2024 | ★ | ★ | ★ |  | ★★ | ★ | ★ | ★ | 8 |
| Guo Y | 2024 | ★ | ★ | ★ | ★ | ★★ | ★ | ★ | ★ | 9 |
| Hamai Y | 2023 | ★ | ★ | ★ | ★ | ★★ | ★ | ★ |  | 8 |
| Hou Y | 2023 | ★ | ★ | ★ | ★ | ★★ | ★ | ★ | ★ | 9 |
| Huai Q | 2023 | ★ | ★ | ★ | ★ | ★★ | ★ | ★ |  | 8 |
| Huang R | 2022 | ★ | ★ | ★ | ★ | ★★ | ★ | ★ |  | 8 |
| Huang X | 2025 | ★ | ★ | ★ |  | ★★ | ★ | ★ |  | 7 |
| Iinuma K | 2021 | ★ | ★ | ★ |  | ★★ | ★ | ★ |  | 7 |
| Ikoma T | 2023 | ★ | ★ | ★ | ★ | ★★ | ★ | ★ | ★ | 9 |
| Inoue H | 2022 | ★ | ★ | ★ | ★ | ★★ | ★ | ★ | ★ | 9 |
| Ishihara H | 2019 | ★ | ★ | ★ | ★ | ★★ | ★ | ★ |  | 8 |
| Jia G | 2023 | ★ | ★ | ★ | ★ | ★★ | ★ | ★ |  | 8 |
| Jiang M | 2020 | ★ | ★ | ★ | ★ | ★★ | ★ | ★ |  | 8 |
| Kadono Y | 2021 | ★ | ★ | ★ |  | ★★ | ★ | ★ | ★ | 8 |
| Katayama Y | 2020 | ★ | ★ | ★ | ★ | ★★ | ★ | ★ | ★ | 9 |
| Knetki-Wróblewska M | 2025 | ★ | ★ | ★ | ★ | ★★ | ★ | ★ |  | 8 |
| Knetki-Wróblewska M | 2023 | ★ | ★ | ★ | ★ | ★★ | ★ | ★ | ★ | 9 |
| Kobayashi K | 2025 | ★ | ★ | ★ | ★ | ★★ | ★ | ★ |  | 8 |
| Ksienski D | 2021 | ★ | ★ | ★ | ★ | ★★ | ★ | ★ |  | 8 |
| Kurashina R | 2022 | ★ | ★ | ★ |  | ★★ | ★ | ★ |  | 7 |
| Kutlu Y | 2023 | ★ | ★ | ★ | ★ | ★★ | ★ | ★ |  | 8 |
| Lei Y | 2025 | ★ | ★ | ★ | ★ | ★★ | ★ | ★ | ★ | 9 |
| Li X | 2024 | ★ | ★ | ★ | ★ | ★★ | ★ | ★ | ★ | 9 |
| Li Y | 2025 | ★ | ★ | ★ | ★ | ★★ | ★ | ★ | ★ | 9 |
| Lin X | 2021 | ★ | ★ | ★ | ★ | ★★ | ★ | ★ |  | 8 |
| Liu J | 2022 | ★ | ★ | ★ | ★ | ★★ | ★ | ★ |  | 8 |
| Liu J | 2019 | ★ | ★ | ★ |  | ★★ | ★ | ★ |  | 7 |
| Lu X | 2022 | ★ | ★ | ★ |  | ★★ | ★ | ★ | ★ | 8 |
| Lu Y | 2025 | ★ | ★ | ★ |  | ★★ | ★ | ★ |  | 7 |
| Luo T | 2025 | ★ | ★ | ★ | ★ | ★★ | ★ | ★ | ★ | 9 |
| Ma Y | 2022 | ★ | ★ | ★ | ★ | ★★ | ★ | ★ | ★ | 9 |
| Matsuki T | 2024 | ★ | ★ | ★ | ★ | ★★ | ★ | ★ |  | 8 |
| Matsuo M | 2022 | ★ | ★ | ★ | ★ | ★★ | ★ | ★ |  | 8 |
| Mesti T | 2023 | ★ | ★ | ★ | ★ | ★★ | ★ | ★ |  | 8 |
| Mildanoglu MM | 2025 | ★ | ★ | ★ |  | ★★ | ★ | ★ | ★ | 8 |
| Muhammed A | 2021 | ★ | ★ | ★ | ★ | ★★ | ★ | ★ | ★ | 9 |
| Numakura K | 2024 | ★ | ★ | ★ | ★ | ★★ | ★ | ★ |  | 8 |
| Olgun P | 2023 | ★ | ★ | ★ | ★ | ★★ | ★ | ★ | ★ | 9 |
| Pan Y | 2021 | ★ | ★ | ★ | ★ | ★★ | ★ | ★ |  | 8 |
| Petrova MP | 2020 | ★ | ★ | ★ | ★ | ★★ | ★ | ★ |  | 8 |
| Pu D | 2021 | ★ | ★ | ★ | ★ | ★★ | ★ | ★ |  | 8 |
| Qi WX | 2023 | ★ | ★ | ★ | ★ | ★★ | ★ | ★ | ★ | 9 |
| Qi WX | 2021 | ★ | ★ | ★ | ★ | ★★ | ★ | ★ |  | 8 |
| Qi Y | 2019 | ★ | ★ | ★ | ★ | ★★ | ★ | ★ |  | 8 |
| Qian X | 2024 | ★ | ★ | ★ |  | ★★ | ★ | ★ |  | 7 |
| Qiu X | 2023 | ★ | ★ | ★ | ★ | ★★ | ★ | ★ |  | 8 |
| Qu Z | 2022 | ★ | ★ | ★ | ★ | ★★ | ★ | ★ | ★ | 9 |
| Rebuzzi SE | 2022 | ★ | ★ | ★ | ★ | ★★ | ★ | ★ | ★ | 9 |
| Russo A | 2020 | ★ | ★ | ★ | ★ | ★★ | ★ | ★ | ★ | 9 |
| Sakai A | 2023 | ★ | ★ | ★ | ★ | ★★ | ★ | ★ |  | 8 |
| Sánchez-Gastaldo A | 2021 | ★ | ★ | ★ | ★ | ★★ | ★ | ★ |  | 8 |
| Shabto JM | 2020 | ★ | ★ | ★ |  | ★★ | ★ | ★ |  | 7 |
| Shang H | 2024 | ★ | ★ | ★ |  | ★★ | ★ | ★ | ★ | 8 |
| Stares M | 2022 | ★ | ★ | ★ |  | ★★ | ★ | ★ |  | 7 |
| Sun Y | 2025 | ★ | ★ | ★ |  | ★★ | ★ | ★ |  | 7 |
| Svaton M | 2018 | ★ | ★ | ★ |  | ★★ | ★ | ★ | ★ | 8 |
| Ucgul E | 2024 | ★ | ★ | ★ |  | ★★ | ★ | ★ |  | 7 |
| Ulas A | 2024 | ★ | ★ | ★ | ★ | ★★ | ★ | ★ | ★ | 9 |
| Su H | 2024 | ★ | ★ | ★ | ★ | ★★ | ★ | ★ | ★ | 9 |
| Diem S | 2017 | ★ | ★ | ★ | ★ | ★★ | ★ | ★ |  | 8 |
| Wan M | 2022 | ★ | ★ | ★ | ★ | ★★ | ★ | ★ |  | 8 |
| Wang JH | 2022 | ★ | ★ | ★ | ★ | ★★ | ★ | ★ |  | 8 |
| Willemsen ACH | 2023 | ★ | ★ | ★ |  | ★★ | ★ | ★ | ★ | 8 |
| Wu J | 2024 | ★ | ★ | ★ | ★ | ★★ | ★ | ★ | ★ | 9 |
| Wu YL | 2022 | ★ | ★ | ★ | ★ | ★★ | ★ | ★ |  | 8 |
| Wu Y | 2022 | ★ | ★ | ★ | ★ | ★★ | ★ | ★ | ★ | 9 |
| Wu Y | 2023 | ★ | ★ | ★ | ★ | ★★ | ★ | ★ |  | 8 |
| Yang X | 2023 | ★ | ★ | ★ | ★ | ★★ | ★ | ★ |  | 8 |
| Yang Y | 2024 | ★ | ★ | ★ | ★ | ★★ | ★ | ★ |  | 8 |
| Yang Z | 2022 | ★ | ★ | ★ | ★ | ★★ | ★ | ★ |  | 8 |
| Yildirim A | 2025 | ★ | ★ | ★ | ★ | ★★ | ★ | ★ |  | 8 |
| Yuan Q | 2024 | ★ | ★ | ★ |  | ★★ | ★ | ★ | ★ | 8 |
| Zhang S | 2025 | ★ | ★ | ★ | ★ | ★★ | ★ | ★ | ★ | 9 |
| Zhang Y | 2022 | ★ | ★ | ★ | ★ | ★★ | ★ | ★ |  | 8 |
| Zhao M | 2022 | ★ | ★ | ★ | ★ | ★★ | ★ | ★ | ★ | 9 |
| Zhu M | 2024 | ★ | ★ | ★ |  | ★★ | ★ | ★ |  | 7 |
| Zhuang TZ | 2025 | ★ | ★ | ★ |  | ★★ | ★ | ★ | ★ | 8 |

Notes: NOS scores range from 0-9. Scores ≥7 indicate high quality.

## Table S3. Meta-regression for OS

| **Variable** | **Level (Contrast)** | **Coefficient** | **SE** | **95% CI** | ***P*-value** |
| --- | --- | --- | --- | --- | --- |
| Cancer Type | Gastric Cancer | -0.043 | 0.272 | -0.576-0.490 | 0.874 |
|  | **HCC** | **0.929** | **0.32** | **0.303-1.556** | **0.004** |
|  | **HNSCC** | **0.89** | **0.357** | **0.191-1.590** | **0.013** |
|  | Lung Cancer | 0.053 | 0.399 | -0.729-0.835 | 0.894 |
|  | NSCLC | 0.42 | 0.271 | -0.112-0.951 | 0.122 |
|  | Other Cancers | 0.48 | 0.283 | -0.073-1.034 | 0.089 |
|  | RCC | 0.353 | 0.298 | -0.231-0.937 | 0.236 |
|  | TNBC | 0.001 | 0.389 | -0.762-0.763 | 0.998 |
|  | Urothelial Carcinoma | 0.344 | 0.317 | -0.277-0.964 | 0.278 |
| Study Design | Retrospective | -0.027 | 0.32 | -0.654-0.600 | 0.933 |
| Region | Europe | -0.305 | 0.175 | -0.649-0.038 | 0.081 |
|  | North America | 0.051 | 0.253 | -0.445-0.547 | 0.84 |
|  | **Other** | **-0.584** | **0.27** | **-1.113--0.055** | **0.03** |
| ICI Class | Camrelizumab | 0.642 | 0.513 | -0.363-1.647 | 0.21 |
|  | Nivolumab | -0.38 | 0.415 | -1.193-0.433 | 0.36 |
|  | Pembrolizumab | -0.402 | 0.45 | -1.285-0.480 | 0.372 |
|  | Mixed/Unknown | -0.303 | 0.393 | -1.073-0.468 | 0.441 |
| **Therapy Line** | **Second or higher** | **-0.426** | **0.206** | **-0.829--0.022** | **0.039** |
|  | **Mixed/Unknown** | **-0.325** | **0.149** | **-0.617--0.034** | **0.029** |
| **Disease Stage** | **Stage III-IV** | **0.568** | **0.241** | **0.096-1.041** | **0.019** |
|  | Stage Ⅱ-IV | 0.285 | 0.406 | -0.511-1.081 | 0.482 |
|  | BCLC B/C | -0.514 | 0.365 | -1.230-0.202 | 0.159 |
|  | BCLC C | -0.486 | 0.631 | -1.723-0.751 | 0.442 |
|  | Advanced | 0.234 | 0.211 | -0.178-0.647 | 0.266 |
|  | **Stage IV** | **0.596** | **0.254** | **0.097-1.094** | **0.019** |
|  | Others | 0.14 | 0.289 | -0.426-0.705 | 0.629 |
| Model Statistics |  | R^2^ = 32.84% |  | QM P = 0.0045 |  |

## Table S4. Meta-regression for PFS

| **Variable** | **Level (Contrast)** | **Coefficient** | **SE** | **95% CI** | ***P*-value** |
| --- | --- | --- | --- | --- | --- |
| Cancer Type | Gastric Cancer | -0.398 | 0.276 | -0.940-0.144 | 0.15 |
|  | HCC | 0.577 | 0.335 | -0.079-1.232 | 0.085 |
|  | HNSCC | 0.542 | 0.469 | -0.378-1.461 | 0.248 |
|  | NSCLC | -0.265 | 0.248 | -0.751-0.221 | 0.285 |
|  | Other Cancers | 0.065 | 0.269 | -0.462-0.592 | 0.81 |
|  | Renal Cell Carcinoma | -0.172 | 0.332 | -0.823-0.479 | 0.604 |
|  | TNBC | -0.282 | 0.364 | -0.996-0.431 | 0.438 |
|  | Urothelial Cancer | 0.226 | 0.451 | -0.658-1.111 | 0.616 |
| Study Design | Retrospective | -0.401 | 0.442 | -1.266-0.465 | 0.364 |
| Region | Asian | 0.126 | 0.341 | -0.542-0.794 | 0.712 |
|  | Europe | -0.003 | 0.342 | -0.673-0.667 | 0.992 |
|  | Other | -0.14 | 0.424 | -0.970-0.691 | 0.742 |
| ICI Class | Camrelizumab | -0.569 | 0.34 | -1.234-0.097 | 0.094 |
|  | Mixed/Unknown | -0.339 | 0.261 | -0.849-0.172 | 0.194 |
|  | Nivolumab | -0.008 | 0.327 | -0.648-0.632 | 0.981 |
|  | Pembrolizumab | -0.264 | 0.408 | -1.064-0.536 | 0.517 |
| **Therapy Line** | **Second or higher** | **-0.623** | **0.234** | **-1.081--0.164** | **0.008** |
|  | **Mixed/Unknown** | **-0.417** | **0.143** | **-0.698--0.137** | **0.004** |
| **Disease Stage** | **Stage III-IV** | **0.532** | **0.229** | **0.085-0.980** | **0.02** |
|  | **BCLC B/C** | **-0.691** | **0.343** | **-1.363--0.020** | **0.044** |
|  | Stage IV | -0.148 | 0.227 | -0.592-0.296 | 0.514 |
|  | Advanced | 0.149 | 0.203 | -0.250-0.547 | 0.464 |
|  | Others | -0.081 | 0.278 | -0.626-0.464 | 0.771 |
| Model Statistics |  | R^2^ = 24.62% |  | QM P = 0.0504 |  |

## Table S5. Sensitivity Analysis of Publication Bias Using the Trim-and-Fill Method for OS and PFS

| Outcome / Model | No. of Studies (k) | Imputed Studies | Pooled HR (95% CI) | *P*-value |
| --- | --- | --- | --- | --- |
| Overall Survival (OS) |  |  |  |  |
| Original Model | 86 | - | 1.87 (1.59–2.20) | < 0.001 |
| Adjusted Model (Trim-and-Fill) | 126 | 40 | 1.105 (0.939–1.300) | 0.231 |
| Progression-Free Survival (PFS) |  |  |  |  |
| Original Model | 72 | - | 1.68 (1.43–1.98) | < 0.001 |
| Adjusted Model (Trim-and-Fill) | 108 | 36 | 1.004 (0.993–1.016) | 0.467 |

## Table S6. Sensitivity Analysis for OS and PFS Stratified by Study Quality Based on NOS Scores

| Outcome / Subgroup | No. of Studies | Hazard Ratio (95% CI) | *P*-value | I^2^ (%) |
| --- | --- | --- | --- | --- |
| Overall Survival (OS) |  |  |  |  |
| All Studies | 86 | 1.01 (1.00–1.01) | 0.0476 | 85.1 |
| High Quality (NOS ≥ 9) | 26 | 1.06 (1.03–1.10) | 0.0003 | 89.1 |
| Lower Quality (NOS < 9) | 60 | 1.00 (1.00–1.01) | 0.1800 | 82.5 |
| Progression-Free Survival (PFS) |  |  |  |  |
| All Studies | 72 | 1.01 (1.00–1.02) | 0.0271 | 83.5 |
| High Quality (NOS ≥ 9) | 26 | 1.06 (1.03–1.10) | 0.0001 | 89.0 |
| Lower Quality (NOS < 9) | 46 | 1.01 (1.00–1.01) | 0.2452 | 77.7 |

## Figure S1


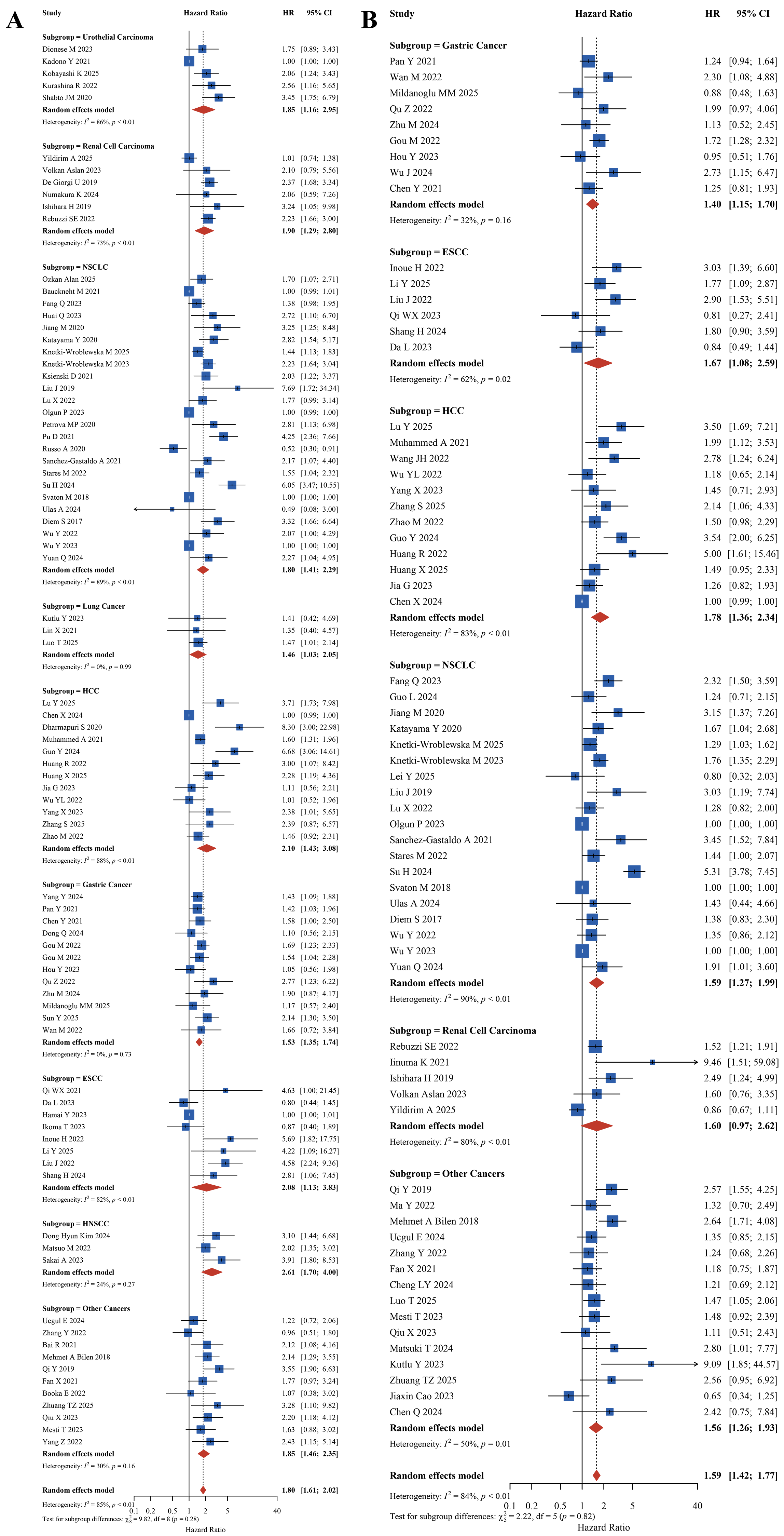


**Figure S1. Forest plots of Cancer Type Subgroup Analysis. (A) Overall Survival (OS). (B) Progression-Free Survival (PFS).**

## Figure S2


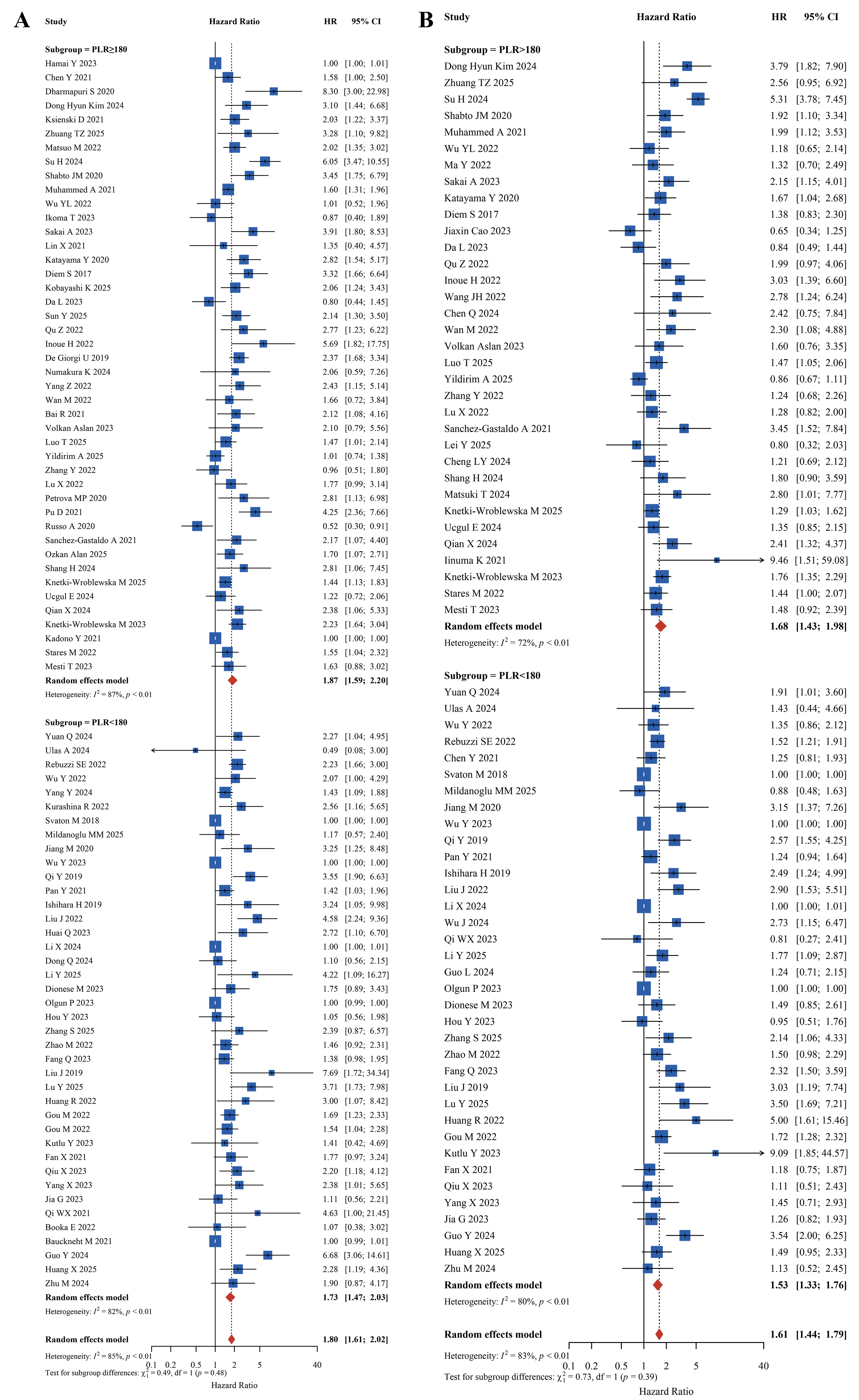


**Figure S2. Forest plots of PLR Cutoff Value** **(A) Overall Survival (OS). (B) Progression-Free Survival (PFS).**

## Figure S3


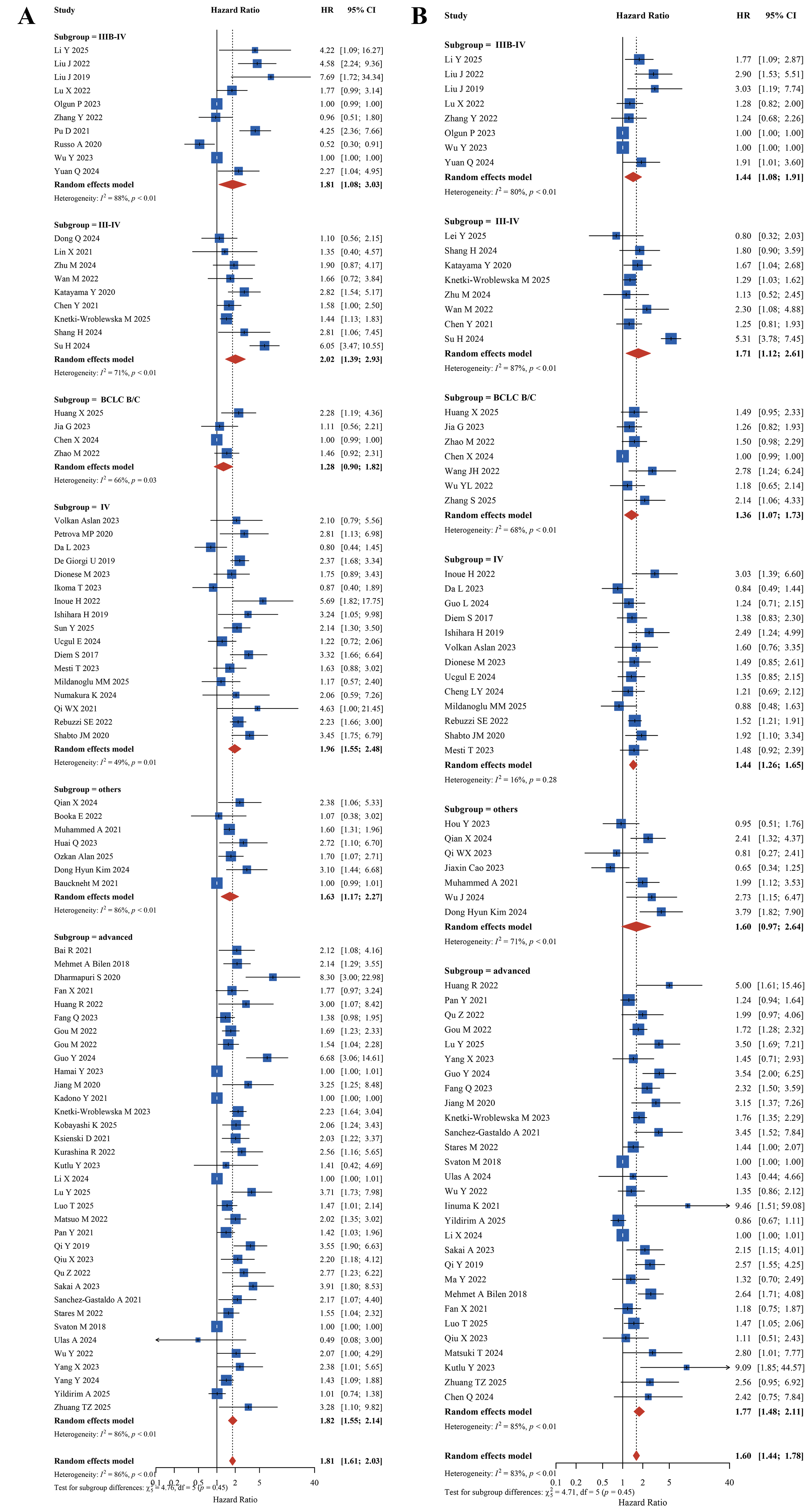


**Figure S3. Forest plots of Tumor Stage Subgroup Analysis. (A) Overall Survival (OS). (B) Progression-Free Survival (PFS).**

## Figure S4


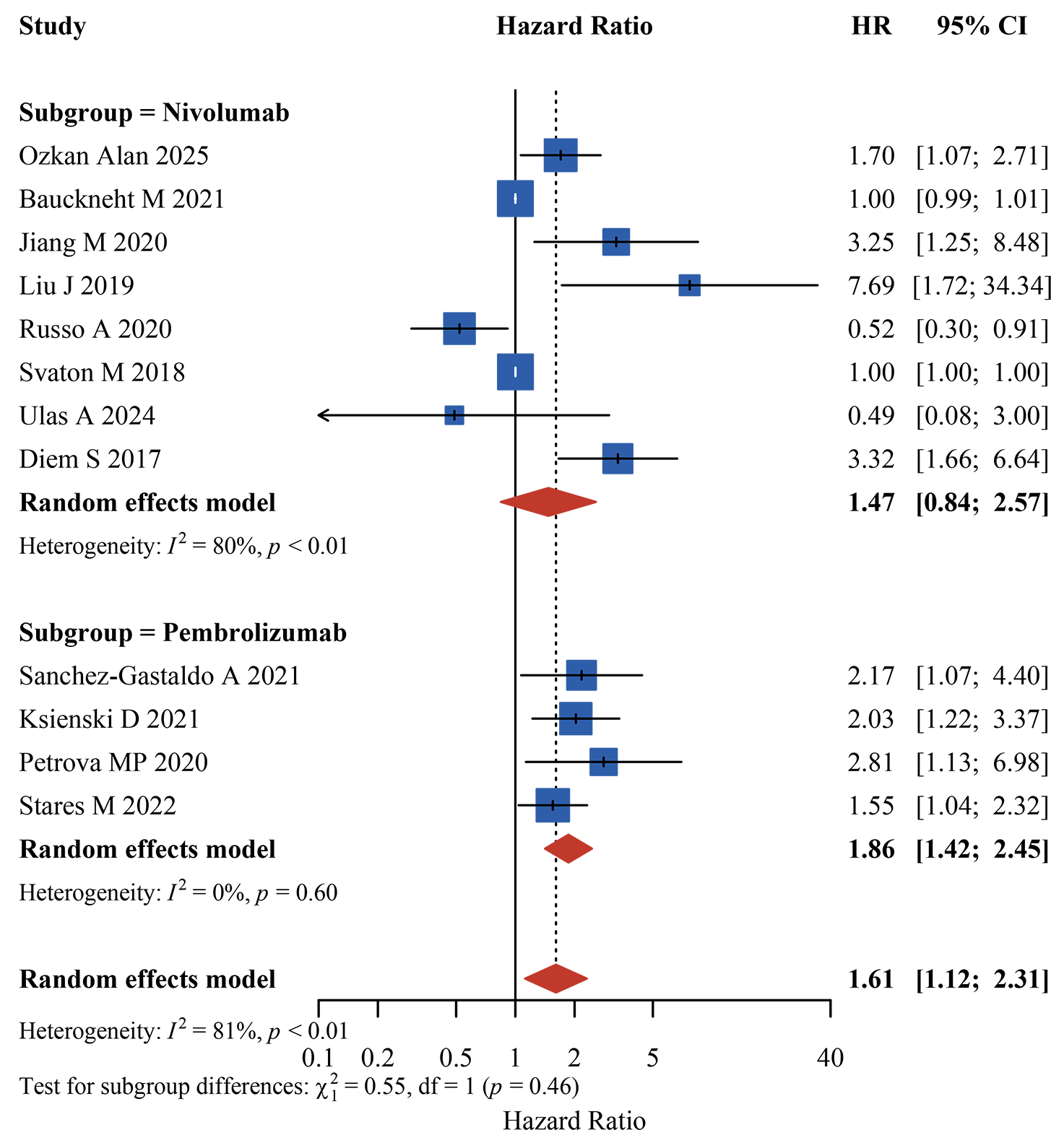


**Figure S4. Forest plots of NSCLC Drug Subgroup Analysis (OS). Overall survival analysis in Non-Small Cell Lung Cancer patients stratified by specific immune checkpoint inhibitors.**

## Figure S5


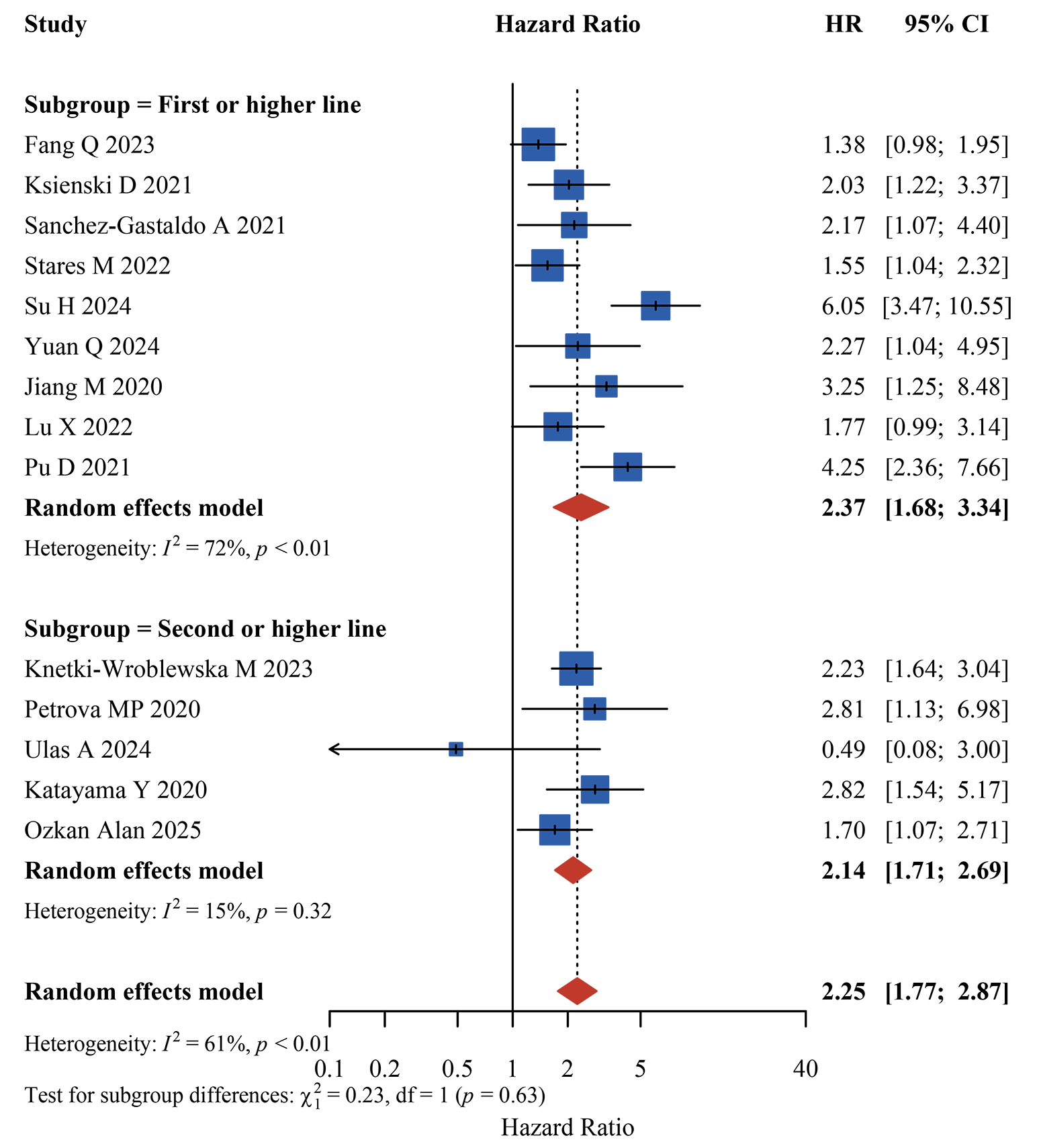


**Figure S5. Forest plots of NSCLC Treatment Line Subgroup Analysis (OS). Overall survival analysis in NSCLC based on treatment line (First-line vs. Second-line or greater).**

## Figure S6


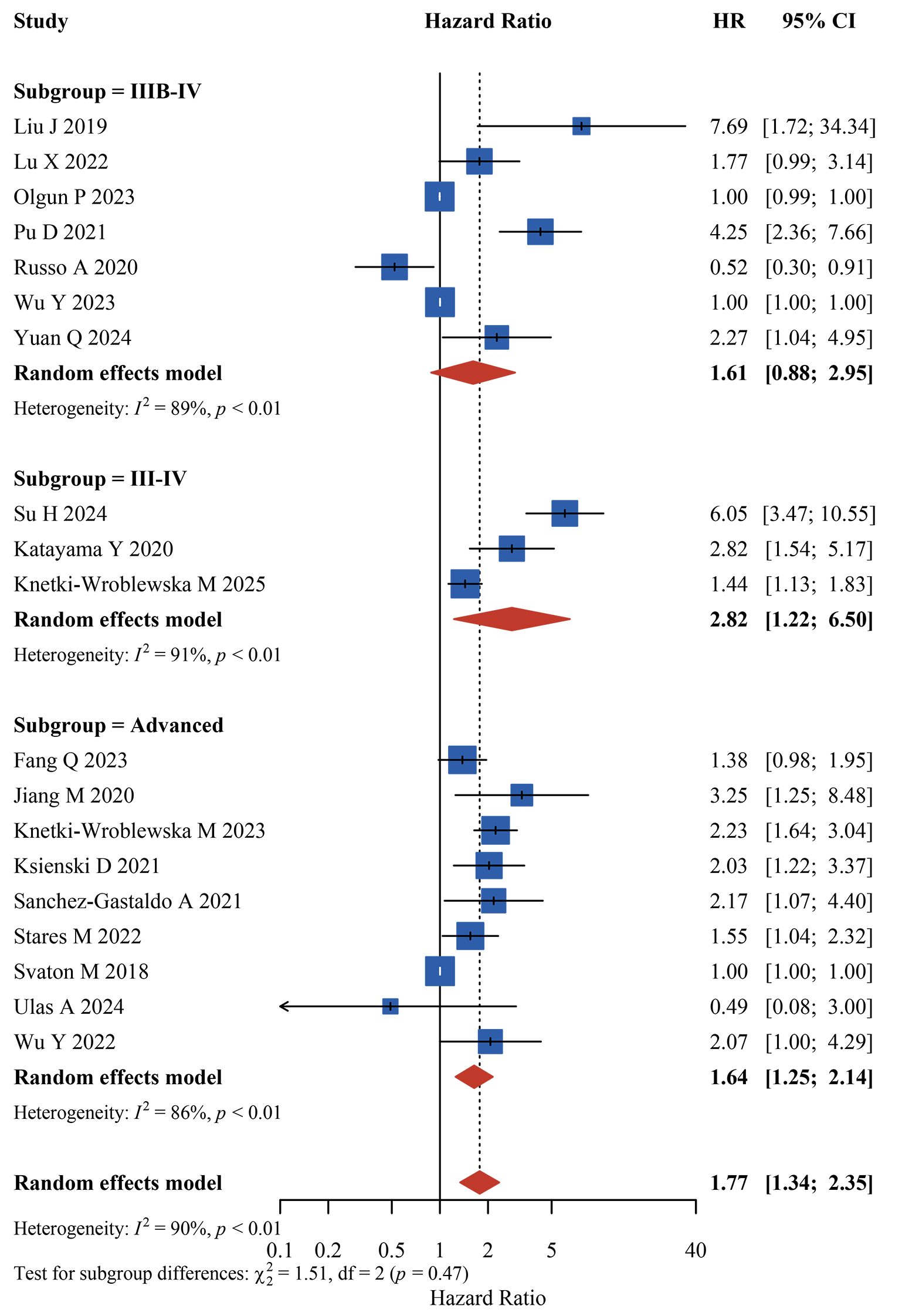


**Figure S6. Forest plots of NSCLC Stage Subgroup Analysis (OS).**

## Figure S7


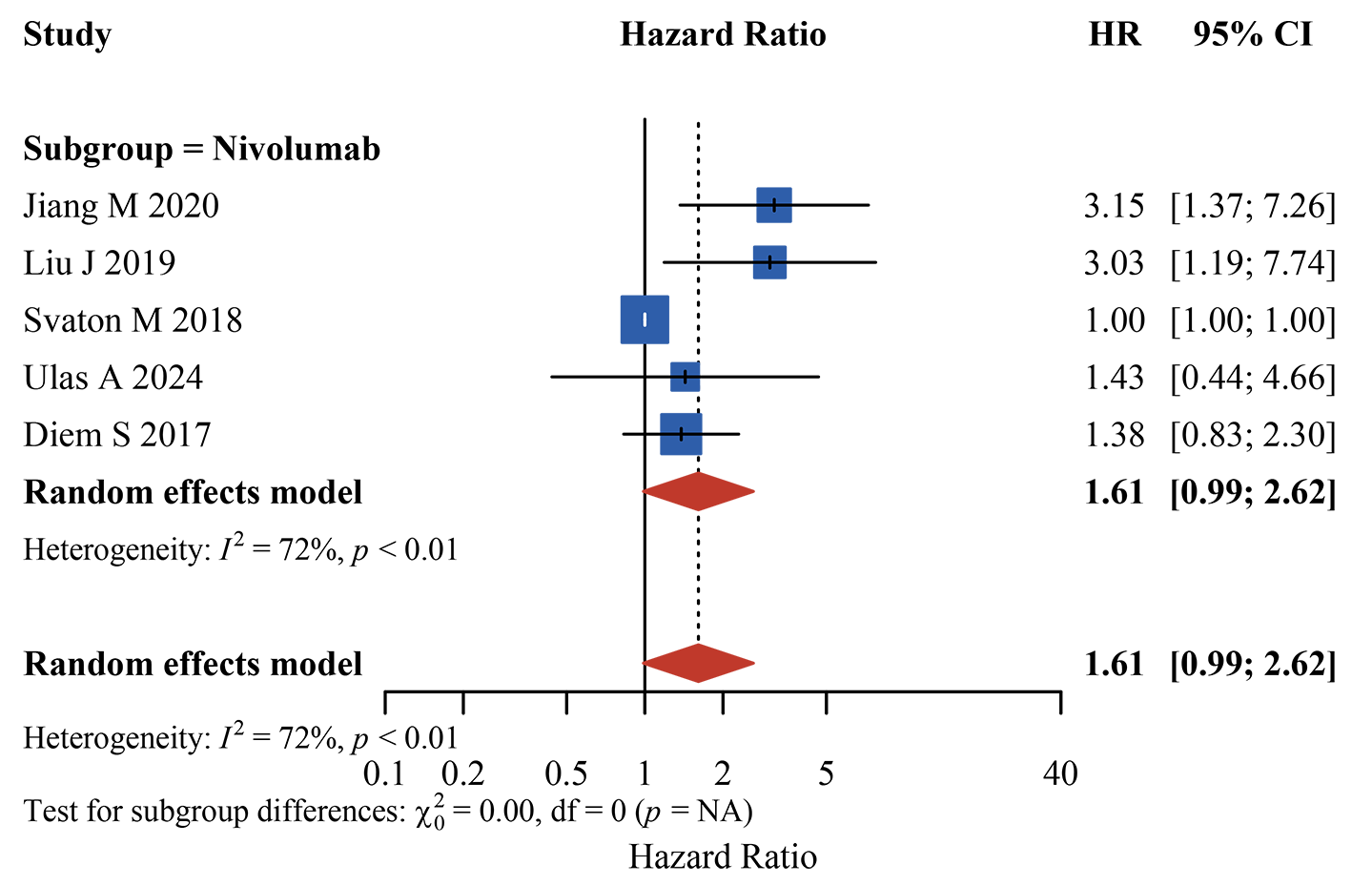


**Figure S7. Forest plots of NSCLC Drug Subgroup Analysis (PFS). Progression-free survival analysis stratified by drug type.**

## Figure S8


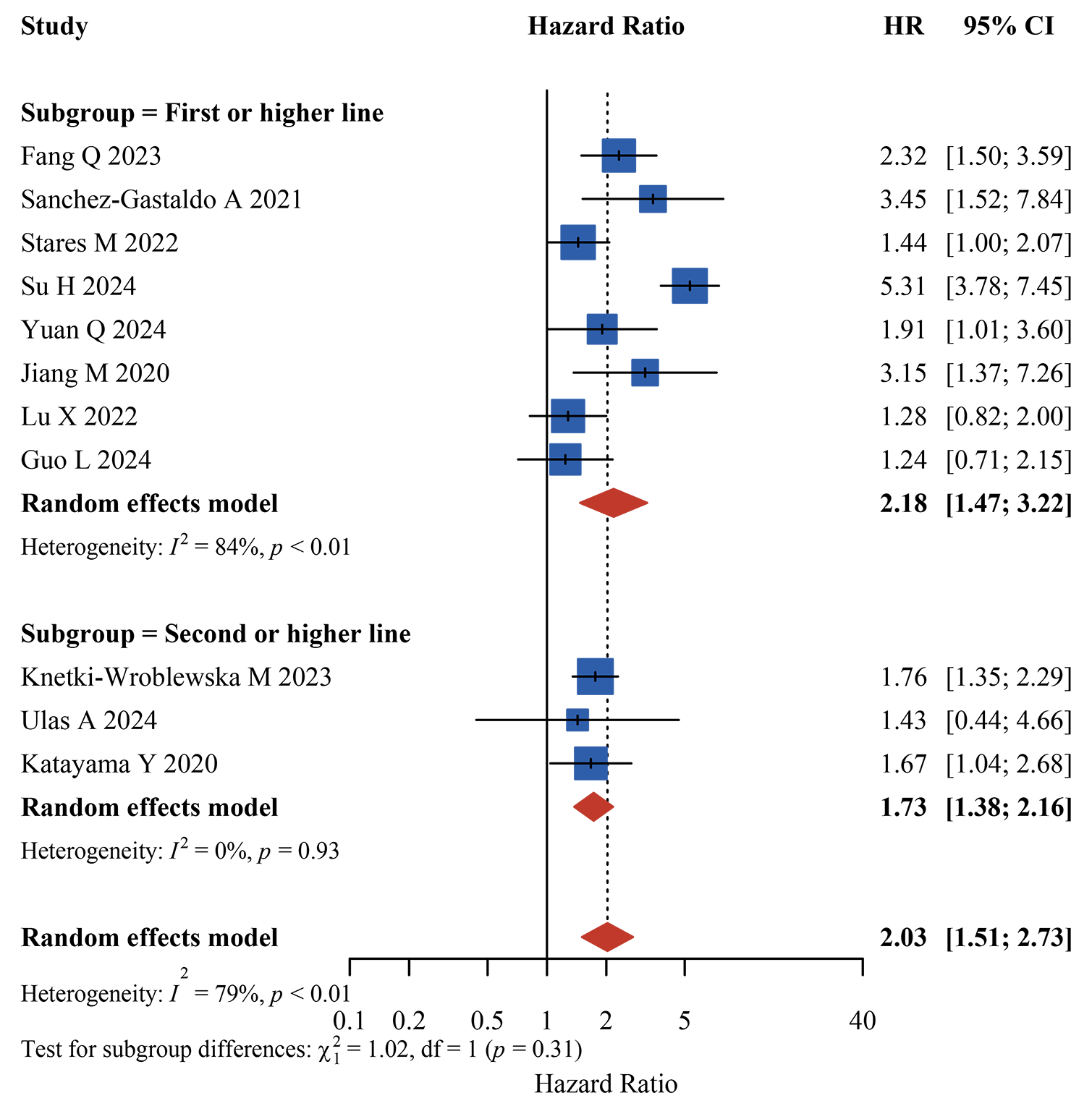


**Figure S8. Forest plots of NSCLC Treatment Line Subgroup Analysis (PFS).**

## Figure S9


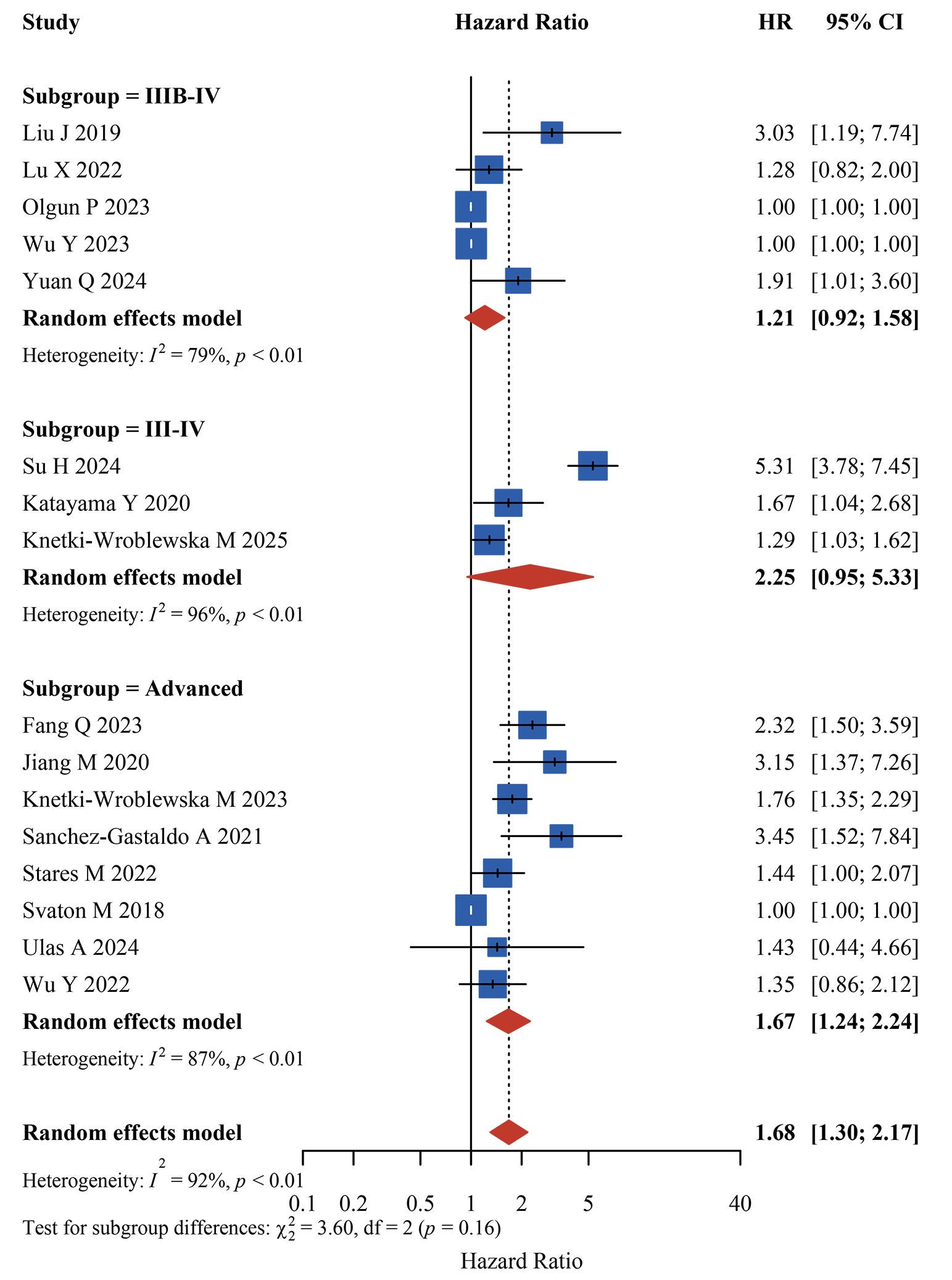


**Figure S9. Forest plots of NSCLC Stage Subgroup Analysis (PFS).**

## Figure S10


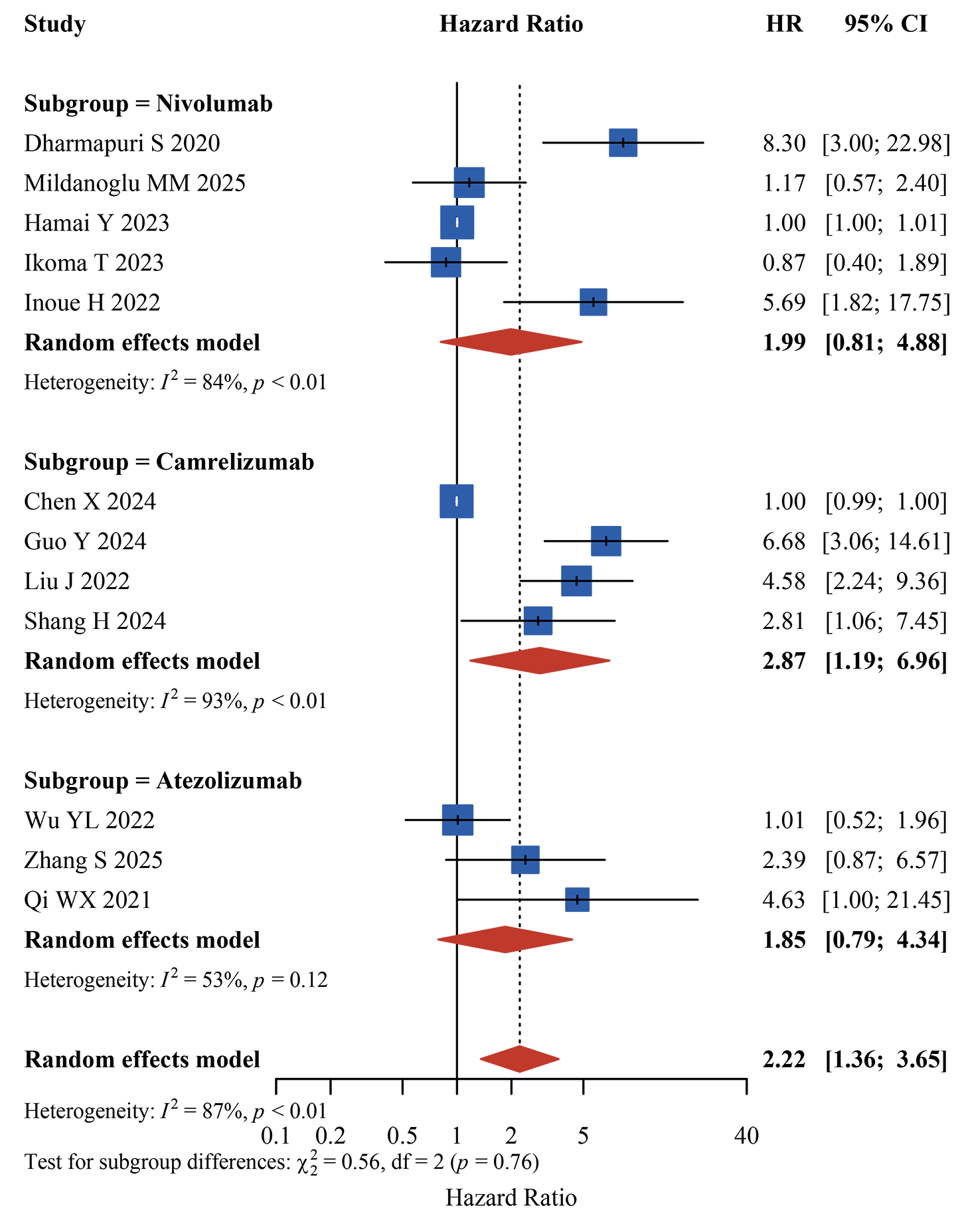


**Figure S10. Forest plots of Gastrointestinal Cancer Drug Subgroup Analysis (OS).**

## Figure S11


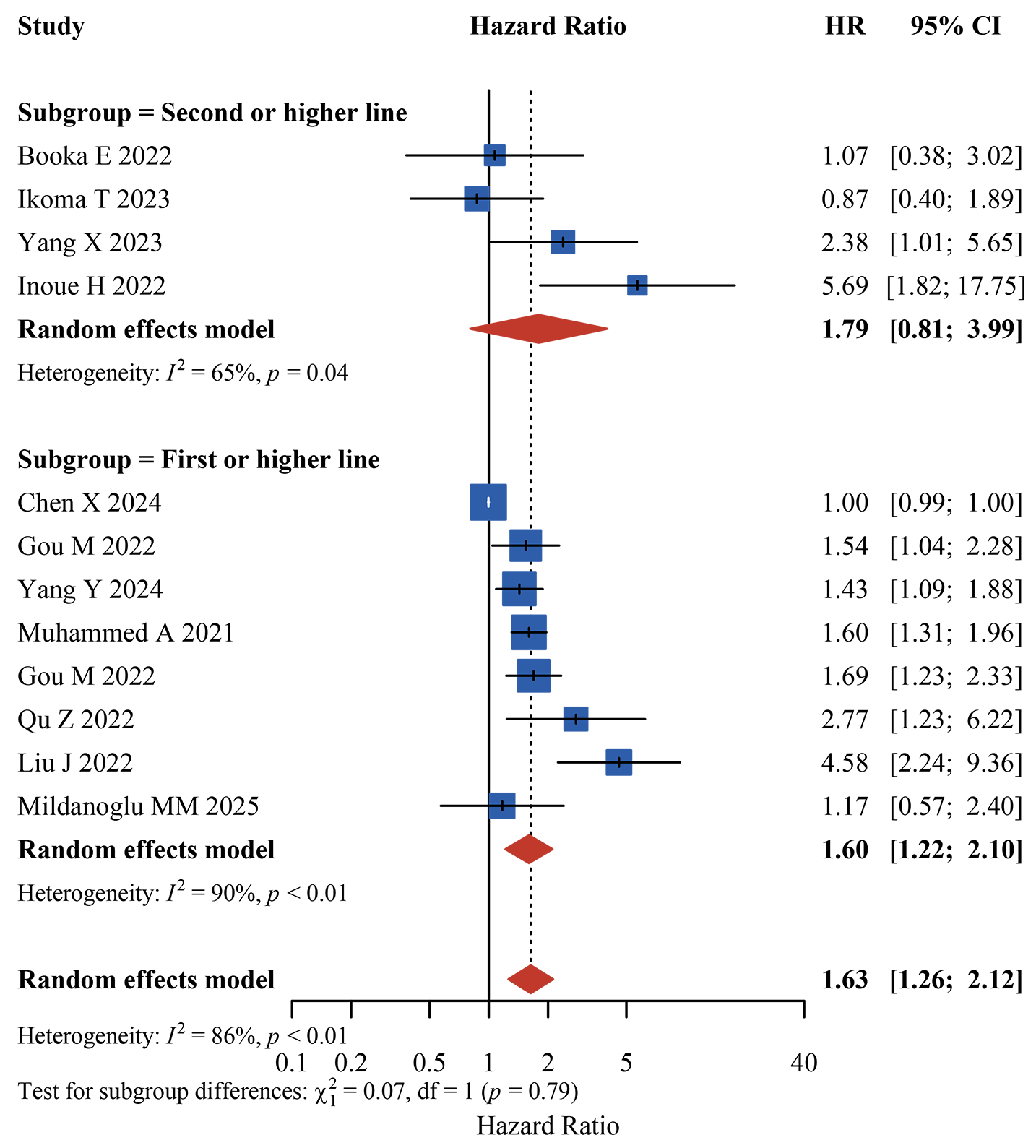


**Figure S11. Forest plots of Gastrointestinal Cancer Treatment Line Subgroup Analysis (OS).**

## Figure S12


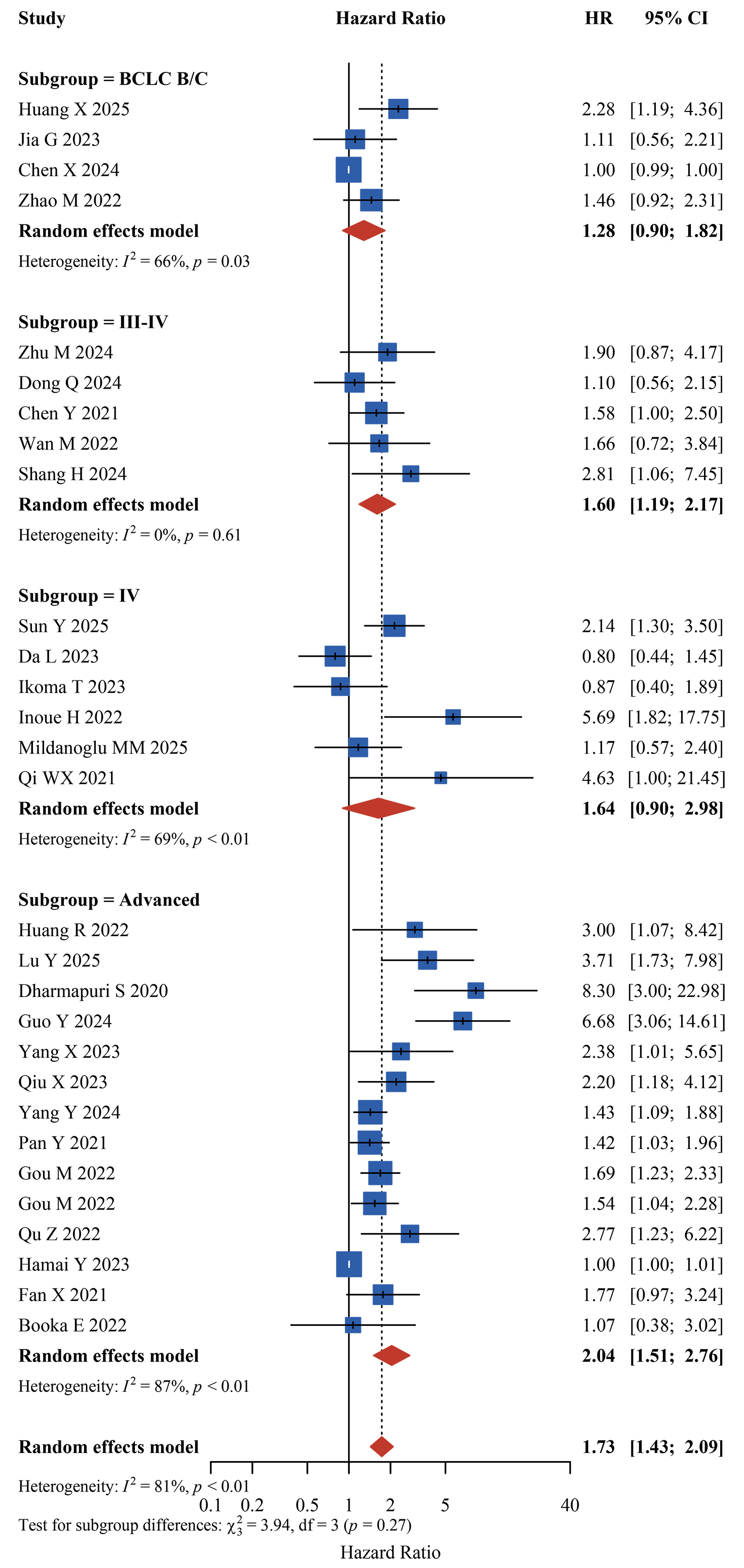


**Figure S12. Forest plots of Gastrointestinal Cancer Stage Subgroup Analysis (OS).**

## Figure S13


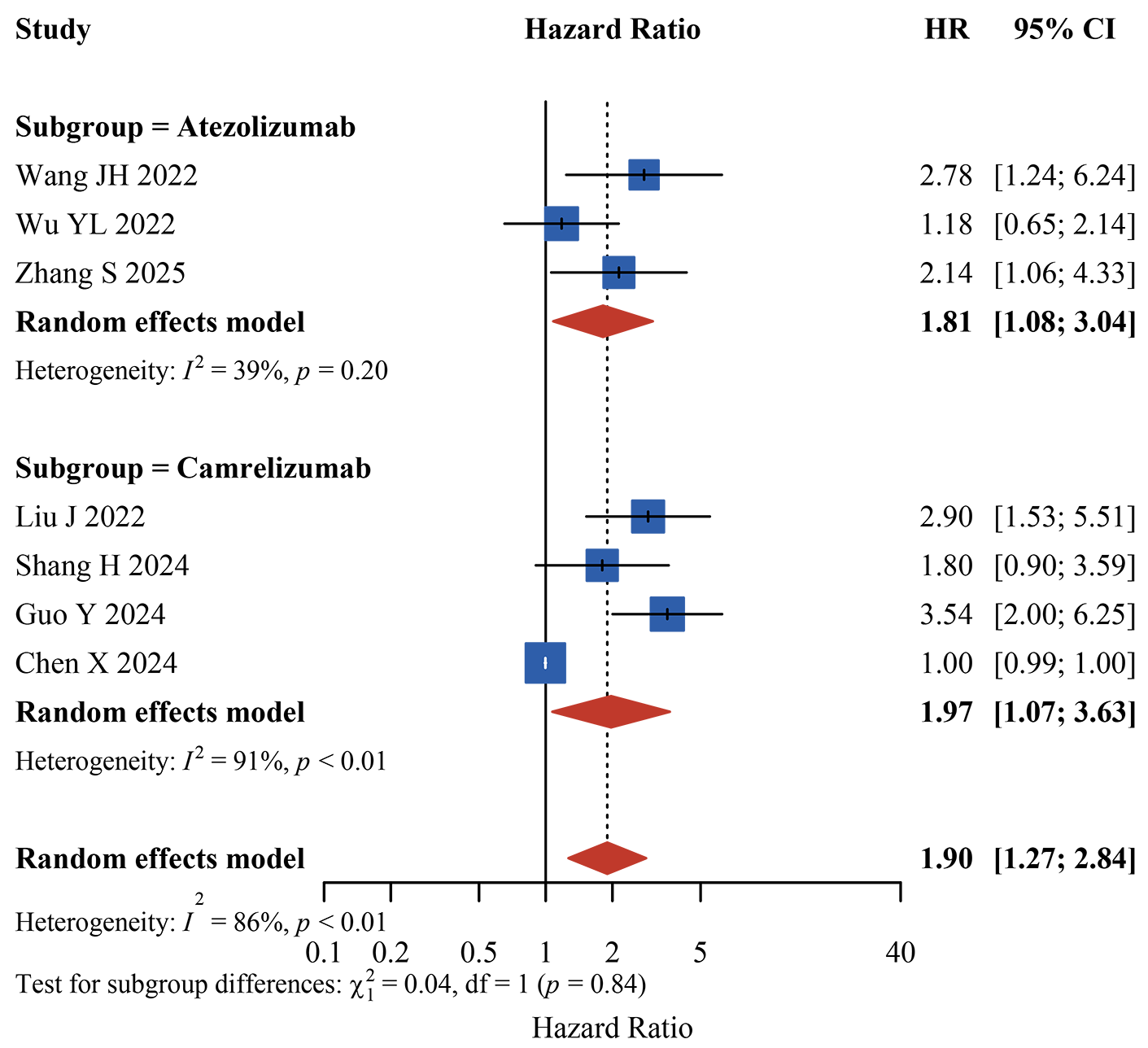


**Figure S13. Forest plots of Gastrointestinal Cancer Drug Subgroup Analysis (PFS).**

## Figure S14


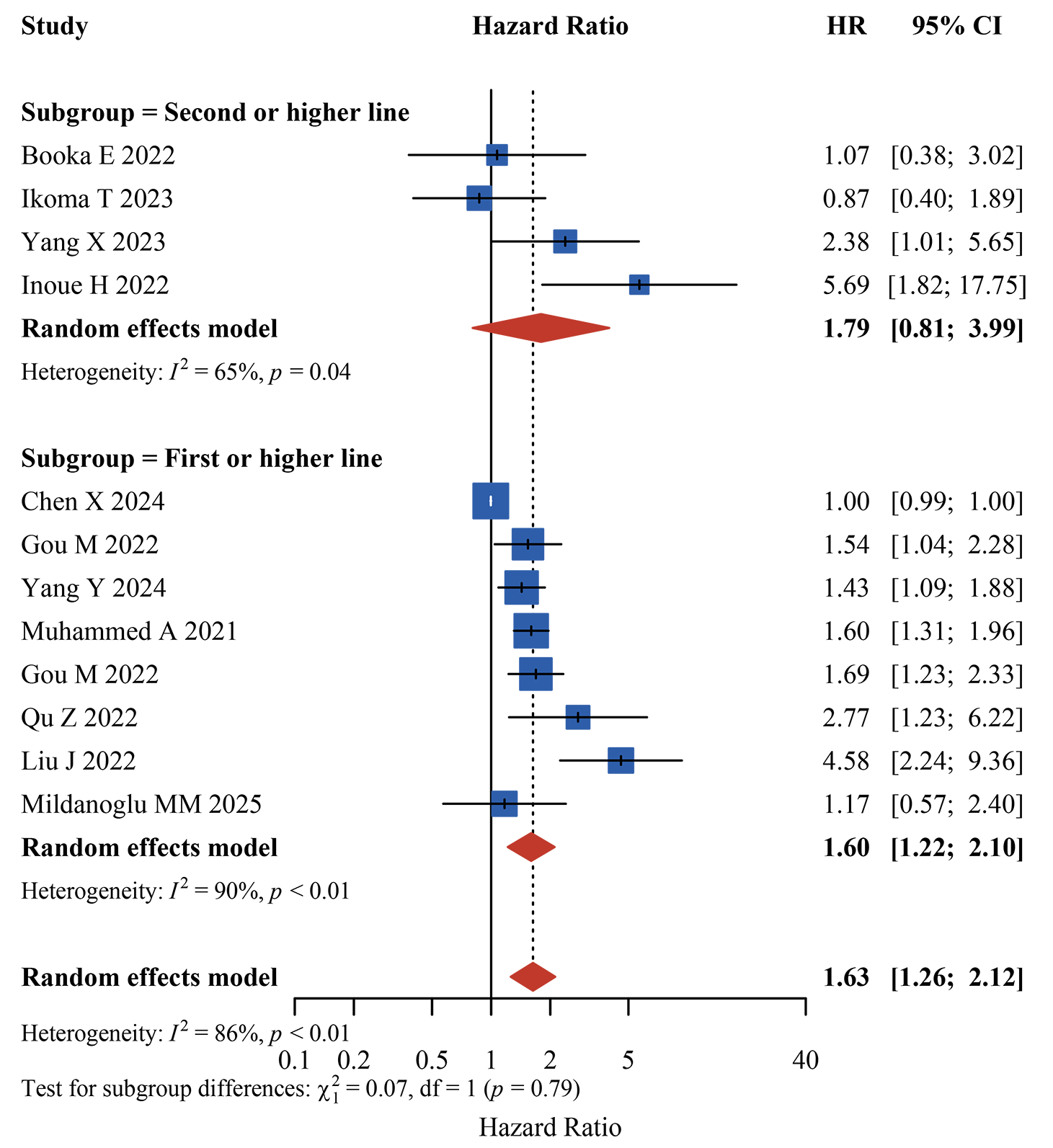


**Figure S14. Forest plots of Gastrointestinal Cancer Treatment Line Subgroup Analysis (PFS).**

## Figure S15


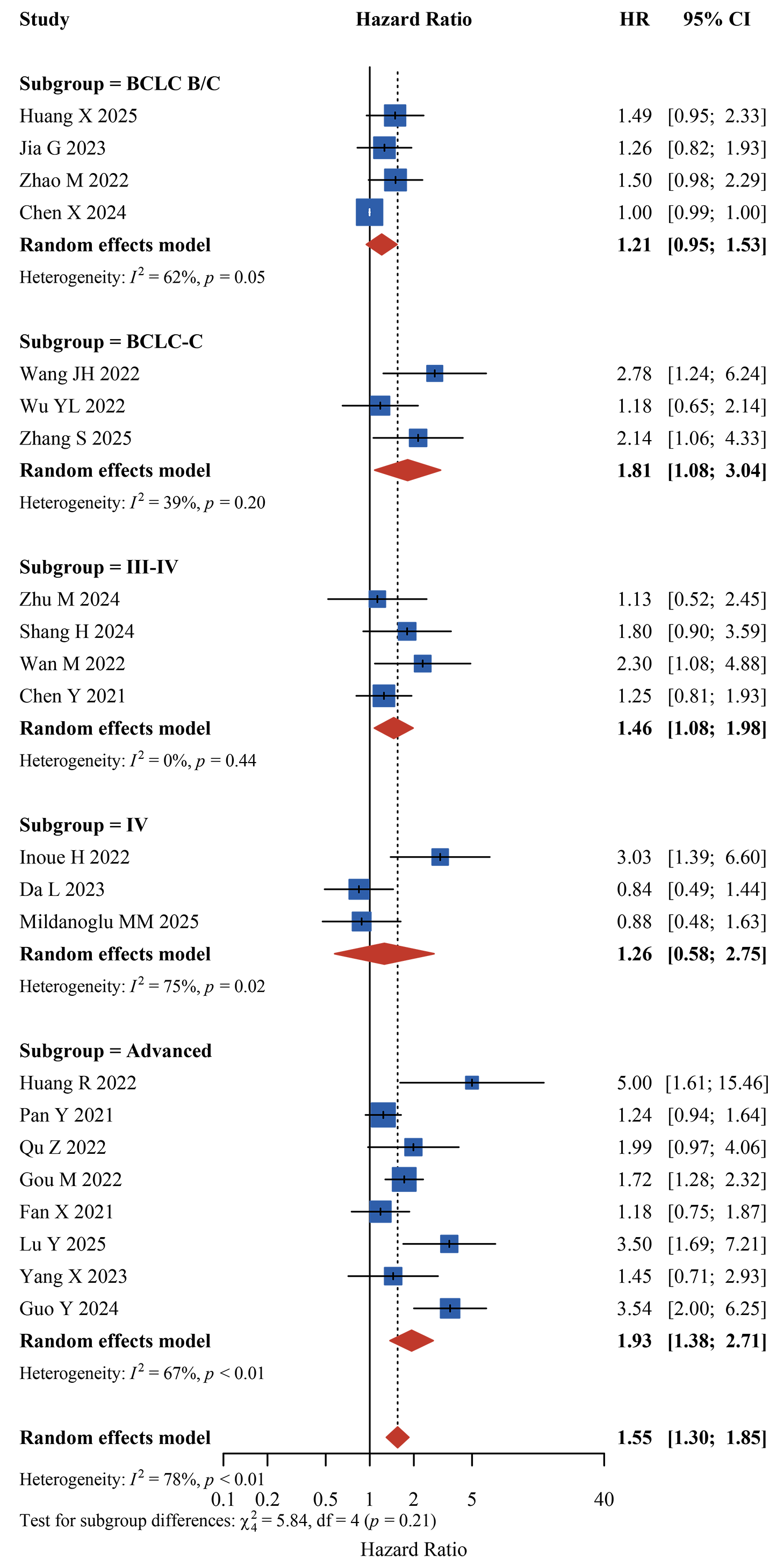


**Figure S15. Forest plots of Gastrointestinal Cancer Stage Subgroup Analysis (PFS).**

## Figure S16


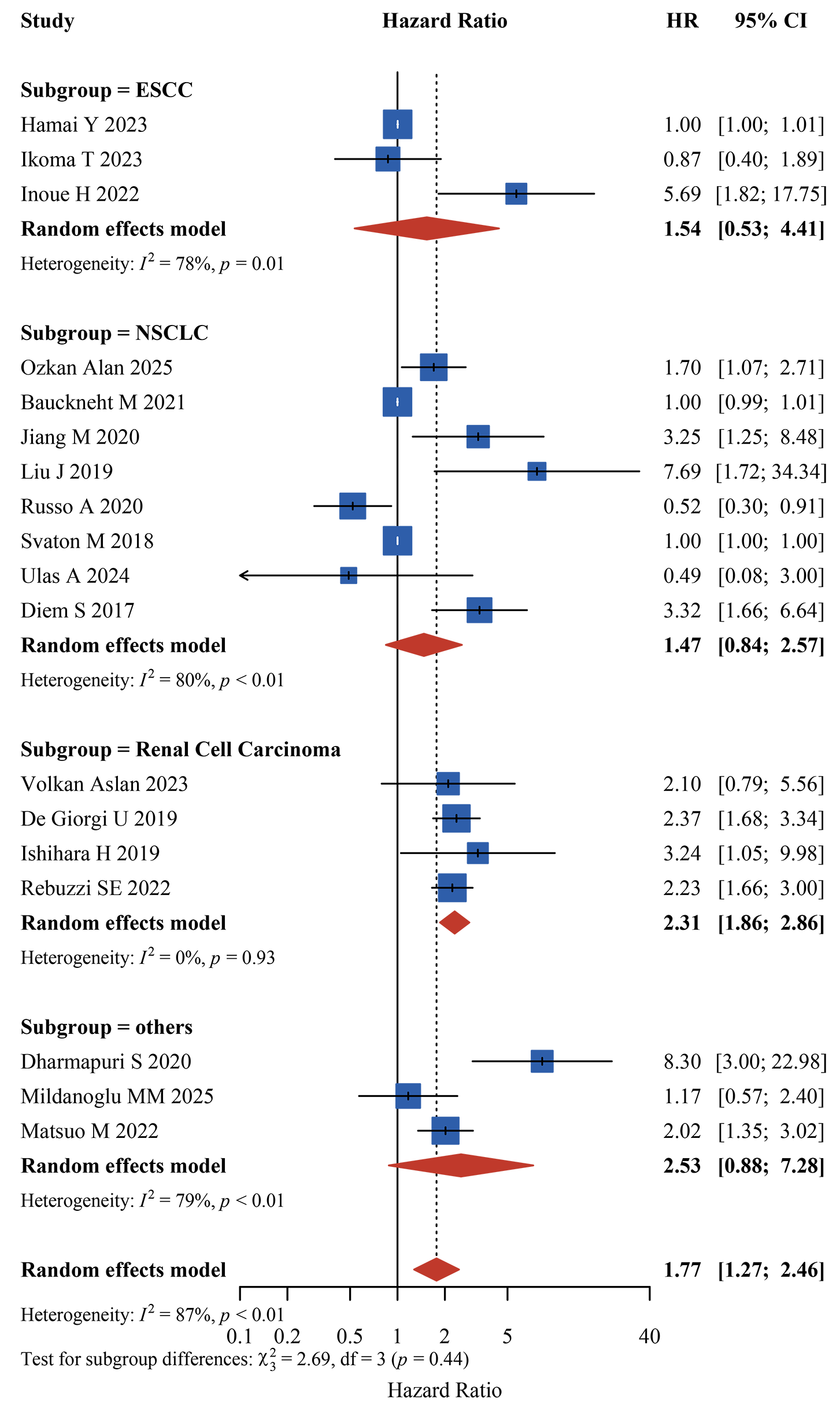


**Figure S16. Forest plots of Cancer Type Subgroup Analysis in Nivolumab-Treated Patients (OS).**

## Figure S17


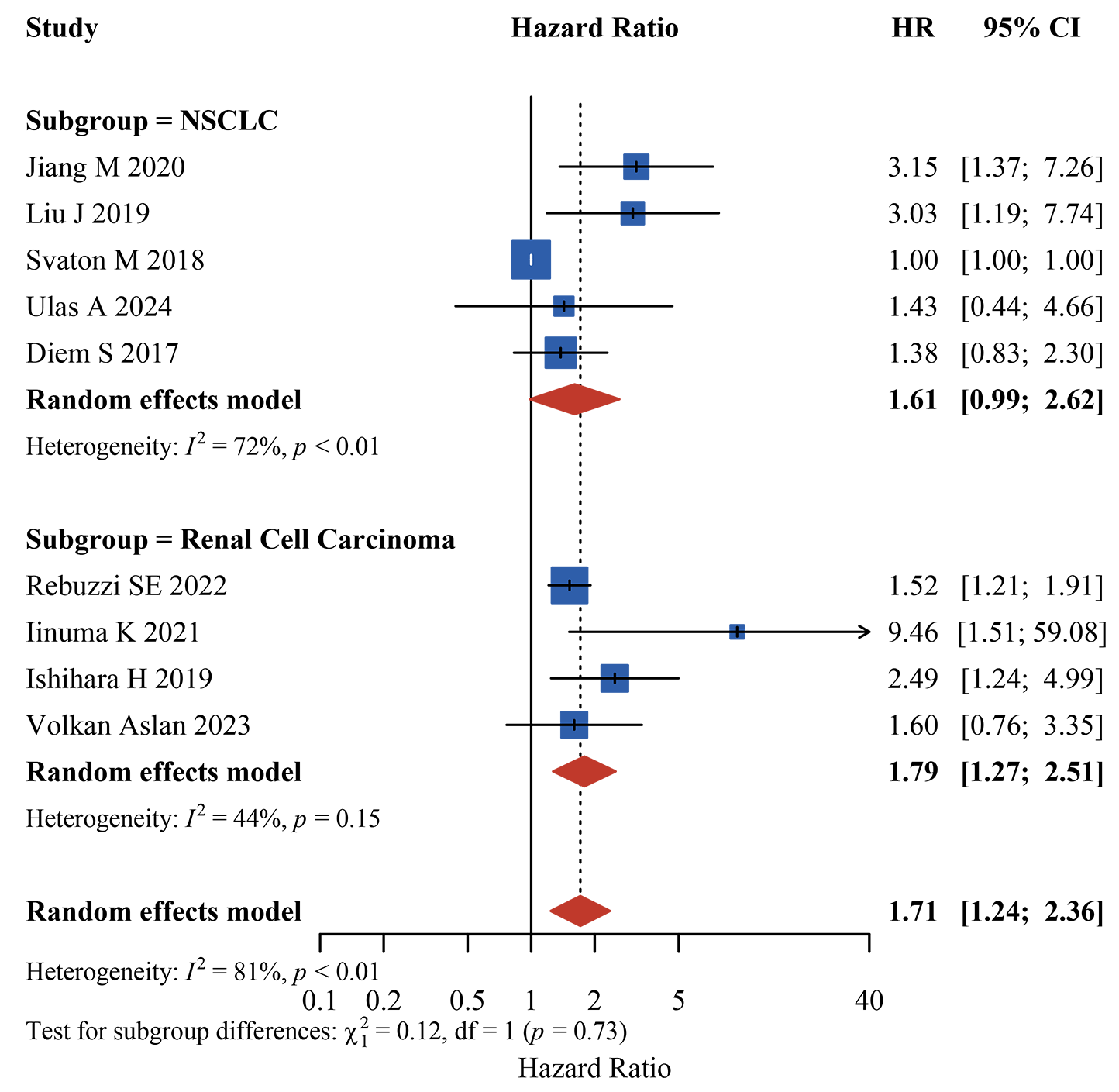


**Figure S17. Forest plots of Cancer Type Subgroup Analysis in Nivolumab-Treated Patients (PFS).**

## Figure S18


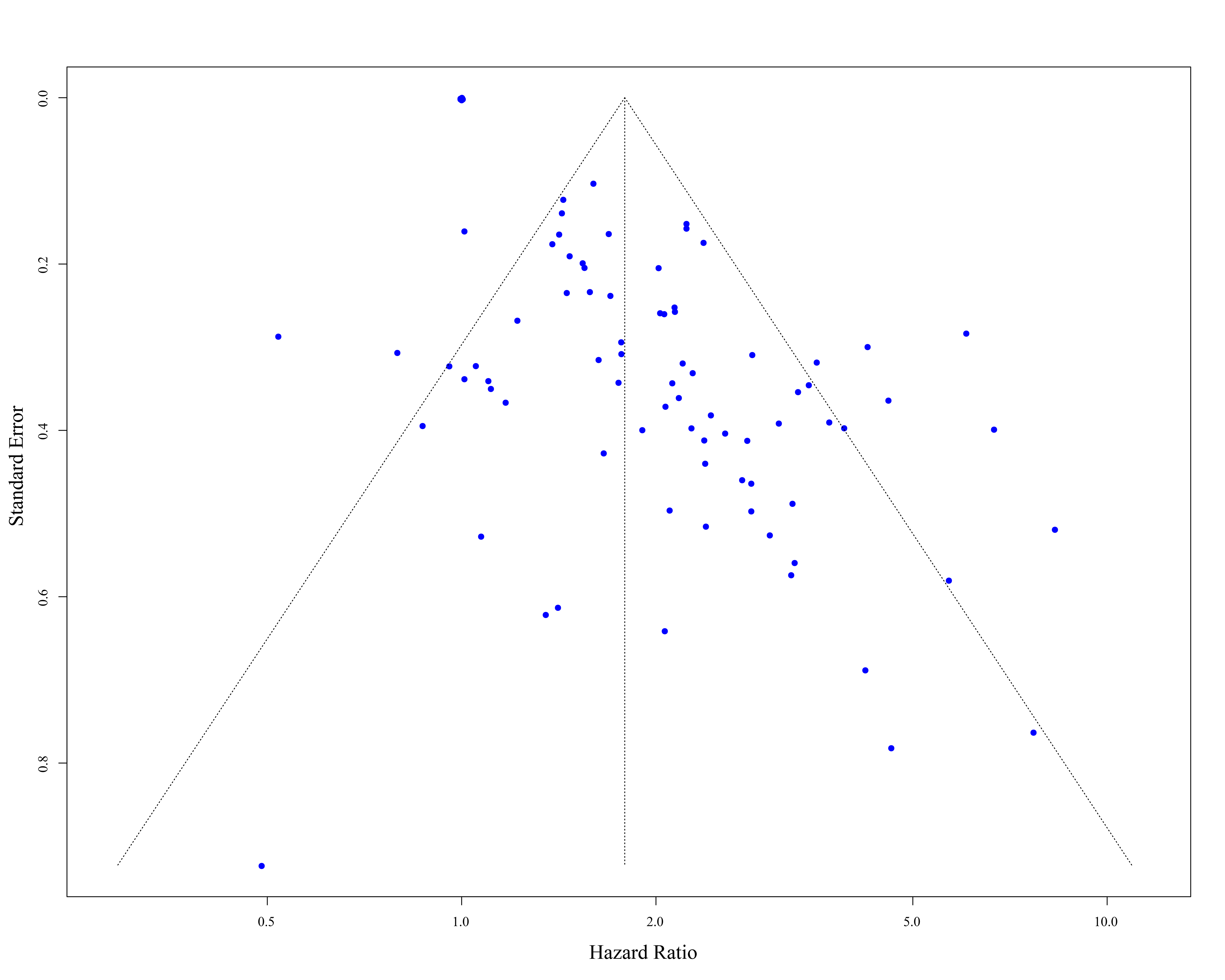


**Figure S18. Funnel Plot of OS Data. Visual inspection of the funnel plot to assess potential publication bias in studies reporting Overall Survival.**

## Figure S19


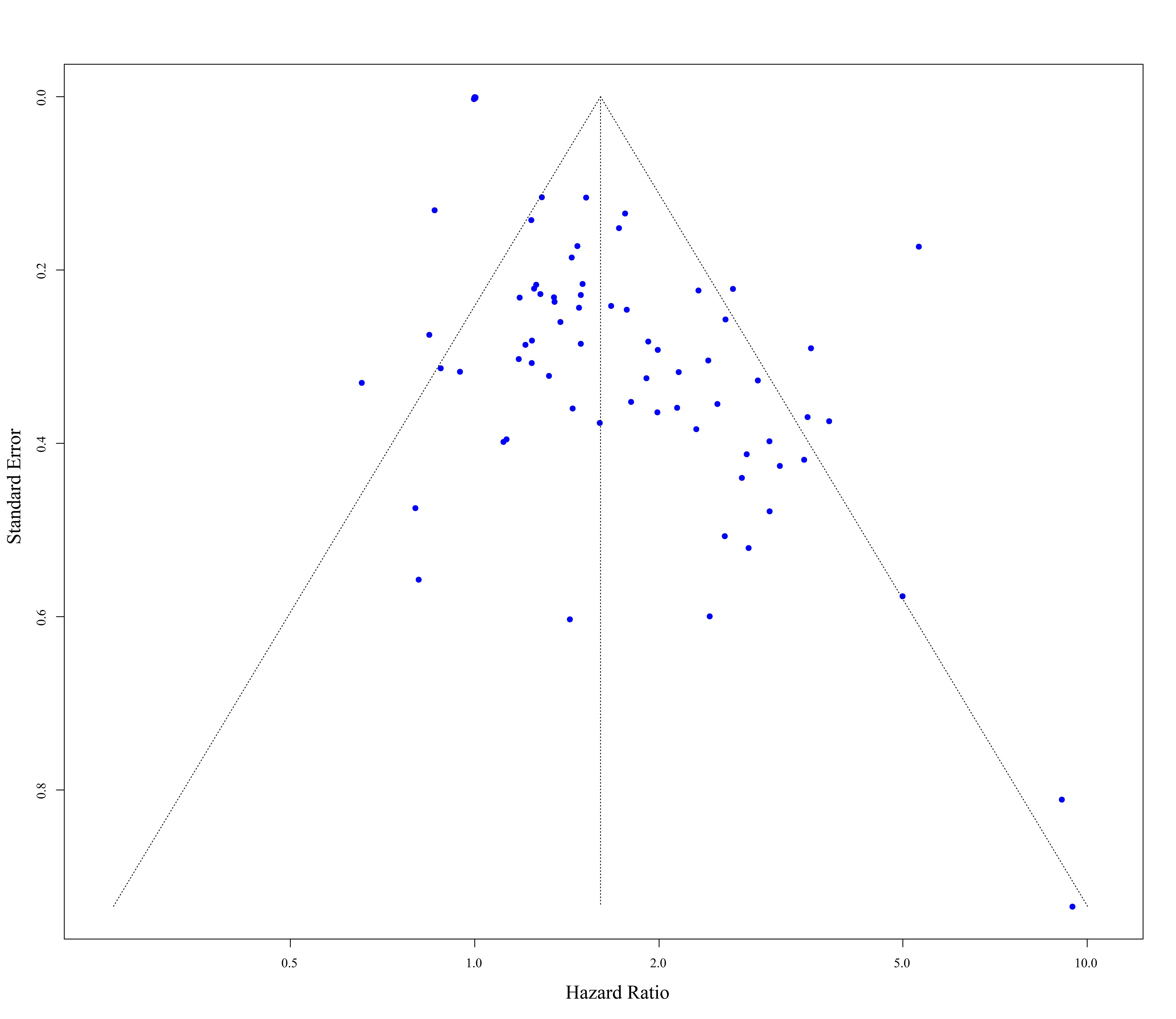


**Figure S19. Funnel Plot of PFS Data. Visual inspection of the funnel plot to assess potential publication bias in studies reporting Progression-Free Survival.**

## Figure S20


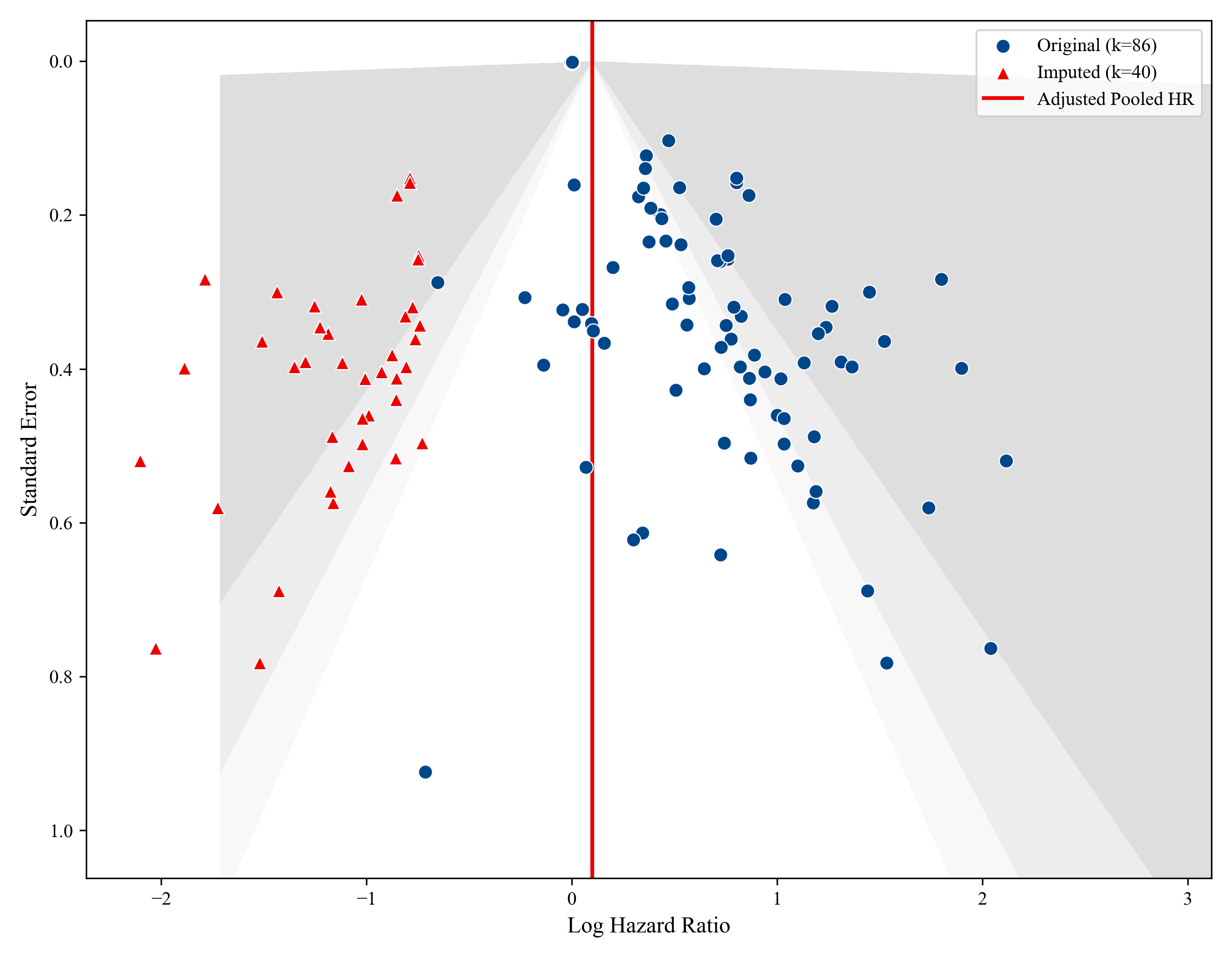


**Figure S20. Funnel plot with trim-and-fill analysis for OS. Blue circles represent observed studies (k=86), and red triangles represent imputed missing studies (k=40). Vertical lines indicate the pooled HRs before (blue) and after (red) adjustment.**

## Figure S21


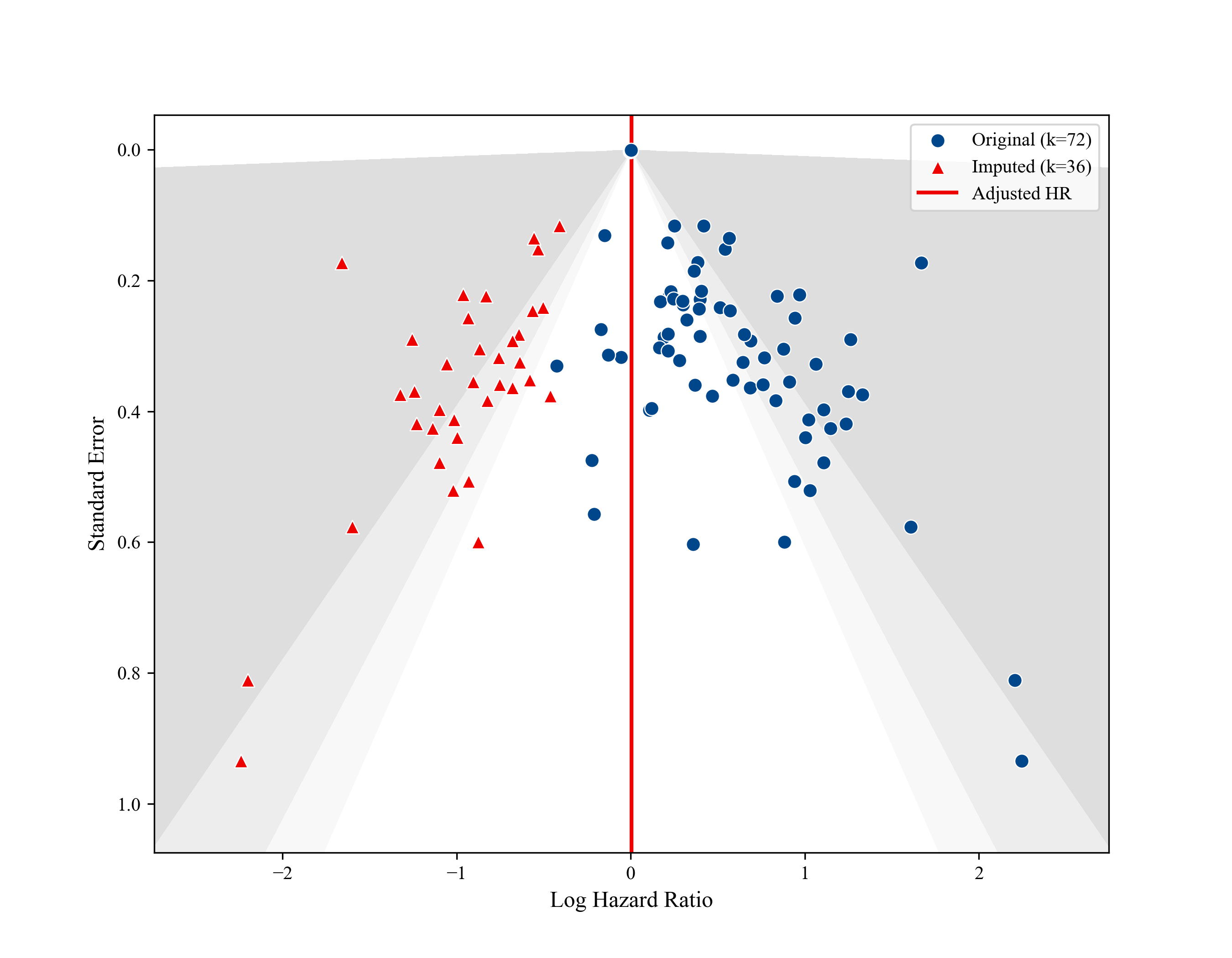


**Figure S21. Funnel plot with trim-and-fill analysis for PFS. Blue circles represent observed studies (k=72), and red triangles represent imputed missing studies (k=36). Vertical lines indicate the pooled HRs before (blue) and after (red) adjustment**

## Figure S22


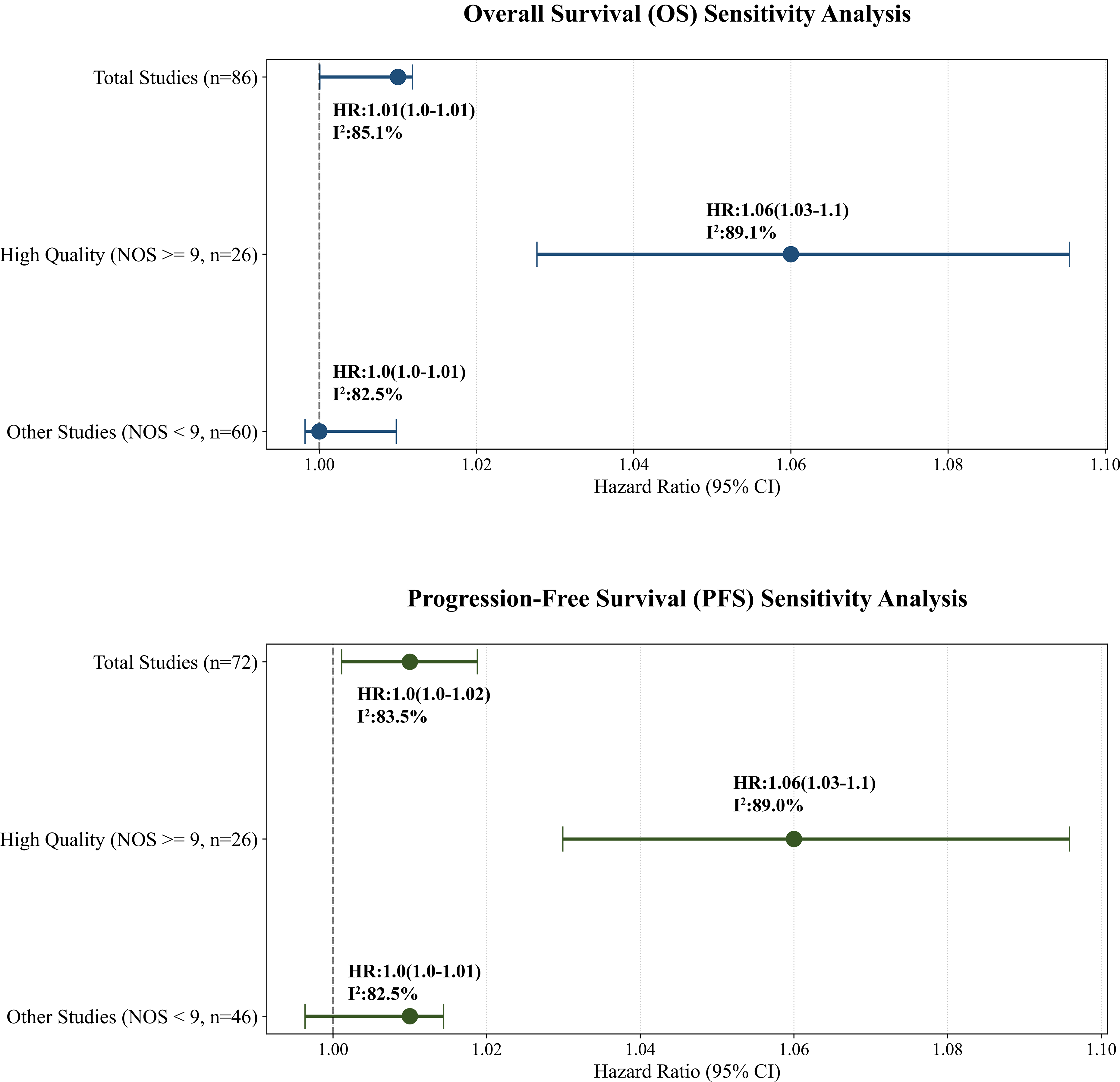


**Figure S22. Forest plots of sensitivity analyses for (A) OS and (B) PFS stratified by study quality.**
